# Arresten, a Collagen-Derived Angiogenesis Inhibitor, Suppresses Invasion of Squamous Cell Carcinoma

**DOI:** 10.1371/journal.pone.0051044

**Published:** 2012-12-05

**Authors:** Mari Aikio, Ilkka Alahuhta, Sini Nurmenniemi, Juho Suojanen, Riitta Palovuori, Susanna Teppo, Timo Sorsa, Carlos López-Otín, Taina Pihlajaniemi, Tuula Salo, Ritva Heljasvaara, Pia Nyberg

**Affiliations:** 1 Oulu Center for Cell-Matrix Research, University of Oulu, Oulu, Finland; 2 Biocenter Oulu and Department of Medical Biochemistry and Molecular Biology, University of Oulu, Oulu, Finland; 3 Department of Diagnostics and Oral Medicine, Institute of Dentistry, University of Oulu, Oulu, Finland; 4 Institute of Dentistry, University of Helsinki, Helsinki, Finland; 5 Departamento de Bioquimica y Biologia Molecular, Facultad de Medicina, Instituto Universitario de Oncologia, Universidad de Oviedo, Oviedo, Spain; 6 Oulu University Hospital, Oulu, Finland; Ottawa Hospital Research Institute, Canada

## Abstract

The turnover of extracellular matrix liberates various cryptic molecules with novel biological activity. Among these are the collagen-derived anti-angiogenic fragments, some of which are suggested to affect carcinoma cells also directly. Arresten is an endogenous angiogenesis inhibitor that is derived from the non-collagenous domain of the basement membrane collagen IV α1 chain. As the mere prevention of tumor angiogenesis leads to hypoxia that can result in selection of more aggressive cell types and reduces the efficacy of chemotherapy, we aimed here to elucidate how arresten influences the aggressive human carcinoma cells. Arresten efficiently inhibited migration and invasion of HSC-3 tongue carcinoma cells in culture and in an organotypic model. Subcutaneous Arr-HSC xenografts grew markedly more slowly in nude mice and showed reduced tumor cell proliferation, vessel density and local invasiveness. In the organotypic assay, HSC-3 cells overproducing arresten (Arr-HSC) showed induction of cell death. In monolayer culture the Arr-HSC cells grew in aggregated cobblestone-like clusters and, relative to the control cells, showed increased expression and localization of epithelial marker E-cadherin in cell-cell contacts. Application of electric cell-substrate impedance sensing (ECIS) further supported our observations on altered morphology and motility of the Arr-HSC cells. Administration of a function-blocking α1 integrin antibody abolished the impedance difference between the Arr-HSC and control cells suggesting that the effect of arresten on promotion of HSC-3 cell-cell contacts and cell spreading is at least partly mediated by α1β1 integrin. Collectively, our data suggest novel roles for arresten in the regulation of oral squamous carcinoma cell proliferation, survival, motility and invasion through the modulation of cell differentiation state and integrin signaling.

## Introduction

Tumor growth does not just depend on carcinoma cells, as interactions between cancer cells, extracellular matrix (ECM) and various cell types in the tumor stoma have a major impact on the disease outcome. The remodeling of tumor stroma during tumorigenesis and the cleavage of basement membrane components results in molecules with novel biological activities [1], [2]. Particularly, collagens IV and XVIII contain cryptic fragments, named arresten, canstatin, hexastatin, tetrastatin, tumstatin and endostatin, which inhibit angiogenesis and tumor growth *via* integrin binding [3]–[15]. Arresten is a 26-kDa fragment derived from the non-collagenous NC1 domain of the basement membrane collagen IV α1 chain [α1(IV)NC1] that efficiently inhibits the proliferation, migration and tube formation of different types of endothelial cells [3], [16]–[18]. *In vivo,* arresten inhibits Matrigel neovascularization [18] and the growth of subcutaneous tumors in mice [3], [16], [18]. It has recently been shown that it also increases apoptosis of endothelial cells by regulating intracellular signaling events. The pro-apoptotic effect of arresten is mediated by reducing the expression of the anti-apoptotic signaling molecules Bcl-2 and Bcl-xL and activating caspase-3/poly (ADP-ribose) polymerase via FAK/p38-MAPK signaling [2], [19]. The production of arresten has recently been linked to the p53 tumor suppressor pathway. p53 was shown to induce an anti-angiogenic program whereby expression of α1(IV) chain is upregulated, stabilized by prolyl-4-hydroxylase and efficiently processed by MMPs to an arresten-containing peptide. This p53-dependent ECM remodeling was suggested to destabilize the vascular collagen IV network and thereby prevent endothelial cell adhesion and migration leading to reduced angiogenesis and tumor growth *in vivo* and *in vitro*. [20].

Tumor cell invasion resulting in metastasis is the main cause of cancer mortality rather than primary tumor growth, and the tumor microenvironment plays a critically important role in this invasion process [21]. In order to metastasize, the tumor cells undergo epithelial-to-mesenchymal transition (EMT)-like events whereby they lose their polarity, and cell-cell and cell-matrix contacts. The acquired mesenchymal, de-differentiated and motile characteristics facilitate cell movement and invasion to novel metastatic locations. The molecular hallmarks of EMT are downregulation of the cell-cell adhesion molecule E-cadherin and upregulation of many mesenchymal markers [22]–[24]. ECM composition and remodeling affect the differentiation state and behavior of tumor cells [25], [26]. For example, increased expression and crosslinking of collagen I and IV are suggested to promote EMT, tumor progression and metastasis [27]–[30]. EMT is a reversible process; during mesenchymal-to-epithelial transition (MET) the cells become again non-motile [22], [31].

The complex interactions between cells and ECM molecules are largely regulated through integrins and other cell surface receptors [32], [33]. Particularly collagen IV has been shown to be the binding substrate of integrins in many cell types, including tumor cells, and its binding to different integrin subtypes may vary depending on its remodeling state [34]. Integrin binding triggers intracellular signaling events that contribute to cancer progression. The pathways leading to EMT *via* regulation of cadherins requires co-operative signals from integrins [32], [33].

As arresten has effects on other cell types in the tumor microenvironment besides endothelial cells [18], we focused here on its impact on highly metastatic human tongue squamous cell carcinoma HSC-3 cell line. By using *in vitro* cell culture assays, organotypic invasion and *in vivo* mouse xenograft models, we show that overexpression of arresten promotes epithelial morphology, and efficiently inhibits proliferation, migration and invasion of carcinoma cells, and induces their apoptosis, leading to suppression of tumor growth and progression.

## Results

### Arresten Inhibits Carcinoma Cell Migration in vitro

After stable transfections, the expression of recombinant arresten was verified in three separate clones of HSC-3 tongue squamous cell carcinoma cells, and also in two MDA-MB-435 breast carcinoma cell clones. By comparison to the parental cells, these stable cell lines showed a substantial increase in arresten expression at mRNA level as ascertained by qPCR (Table S1). More importantly, a ∼29 kDa Flag-tagged arresten was detected by Western blotting in the conditioned medium (CM) collected from Arr-HSC and Arr-MDA cells (Figure S1A–B). The following experiments were performed using Ctrl-HSC(1) and Arr-HSC(1) (Figure S1) clones unless otherwise stated.

**Figure 1:**
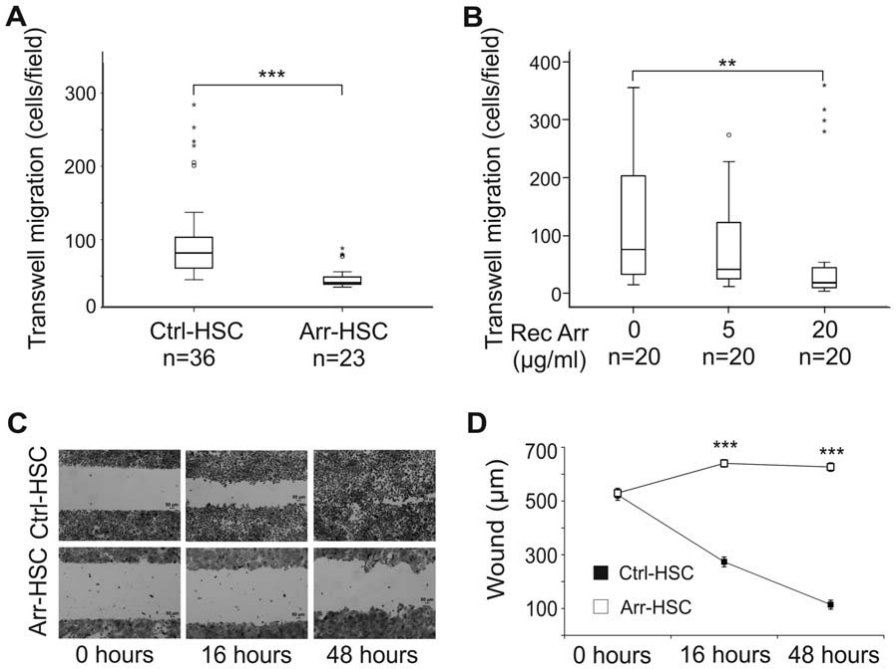
Arresten inhibits migration of HSC-3 cells. **A.** 30 000 Ctrl-HSC and Arr-HSC cells were allowed to migrate through Transwell inserts and the number of migrated cells was counted under a microscope at 50×magnification. Mann-Whitney U-test, ***p<0.001, (n = total number of fields analyzed, 2–4 fields per Transwell insert). **B.** 30 000 HSC-3 cells were allowed to migrate through Transwell inserts in the presence of human recombinant purified arresten (5 and 20 µg/ml) and the number of migrated cells was counted as described above. Mann-Whitney U-test, **p<0.01, (n = total number of fields analyzed, 3–5 fields per Transwell insert). **C.** Scratch wound healing assay with Ctr-HSC and Arr-HSC clones in which the closure of the wound was measured at 0, 16 and 48 h. Scale bar 50 µm**.** **E.** Quantification of scratch wound healing in the Ctrl-HSC and Arr-HSC clones. Mann-Whitney U-test, ***p<0.001, (n = 70 fields at 0, 16 and 48 h per clone).

To study the effects of arresten on carcinoma cells, we first performed Transwell migration experiments and found that the Arr-HSC cells migrated significantly less than the control cells (p<0.001) (Figure 1A). The addition of exogenous human recombinant arresten had a similar inhibitory and dose-dependent effect on Ctrl-HSC cell migration in Transwell assay (Figure 1B). Furthermore, the Arr-HSC clones showed a clear non-migratory phenotype in the scratch wound healing assay, whereas the control cells almost closed the wound within 48 h (Figure 1C–D, Figure S2A and S2C). Also the Arr-MDA breast carcinoma cells were statistically less motile than the Ctrl-MDA cells in the wound healing assay (Figure S2B and S2D). HSC-3 cell proliferation, measured by BrdU incorporation into the DNA-synthesizing cells, was not affected by the overexpression of arresten within 24 h (Figure S3A), but a reduced number of viable arresten cells was observed in the MTT assay in a longer experimental set-up (68 h) in monolayer culture (p = 0.001) (Figure S3B).

To confirm that the observed significant change in the Arr-HSC cell motility was not due to an artifact of overexpression, but rather to the secretion of arresten into the culture medium we collected CM from the Arr-HSC cells, transferred it to Ctrl-HSC cells and measured the effect on cell migration by Transwell assay. The migration of Ctrl-HSC cells decreased approximately 40% in the presence of conditioned Arr-HSC medium (p<0.001) (Figure S4A). To verify that the secreted arresten did not become degraded during the co-culture period, we collected CM for Western blot analysis at various time points of culture. This analysis showed that no protein degradation occurred during the 72 h culture period (Figure S4B).

### Arresten Reduces Tumor Vasculature and Suppresses Growth and Invasion of HSC-3 Xenografts

**Figure 2:**
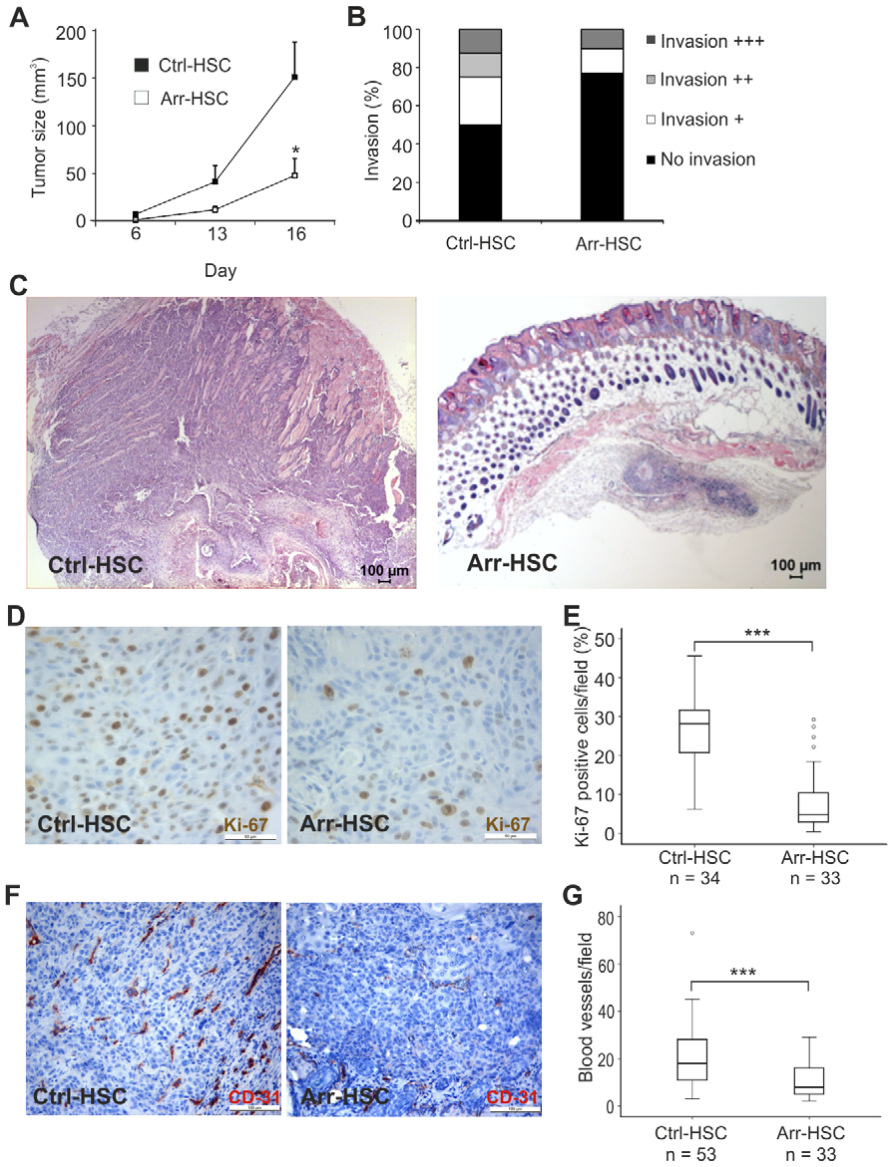
Effects of arresten on HSC-3 xenografts. **A.** One million Ctrl-HSC and Arr-HSC cells were injected subcutaneously into the flanks of nude mice and tumor growth was monitored over 16 days. Students t-test, *p<0.05, (n = 10 mice per group). **B.** Local invasiveness of the tumors. **C.** Representative hematoxylin-eosin stainings of HSC-3 xenografts. Scale bar 100 µm. **D–E.** HSC-3 xenografts were stained for the proliferation marker Ki-67 (brown) and the cell proliferation was defined as a percentage of Ki-67-positive cells among the total number of carcinoma cells per microscopic field (400×magnification; n = number of fields analyzed, 3–5 fields per xenograft). Scale bar 50 µm. **F–G.** The tumor blood vessels were stained with a CD31 antibody and counted under a microscope (200×magnification; n = number of fields analyzed, 3–5 fields per xenograft). Mann-Whitney U-test, ***p<0.001. Scale bar 100 µm.

Ctrl-HSC or Arr-HSC carcinoma cells were injected subcutaneously into nude mice and tumor growth was monitored for 16 days. The Arr-HSC tumors grew significantly more slowly than the control tumors (Figure 2A). In addition, some differences in local tumor invasion were noted between Arr-HSC and Ctrl-HSC xenografts upon histopathological examination (Figure 2B–C). Most (∼80%) of the arresten tumors had not invaded into the surrounding tissue, whereas half of the control tumors showed at least minor score of invasiveness (Figure 2B). Our observation of the less invasive phenotype of Arr-HSC xenografts was supported by an *in vitro* experiment, where the Arr-HSC cells invaded less through Matrigel than the Ctrl-HSC cells (Figure S5). Immunostaining of HSC-3 xenografts for Ki-67 revealed almost 70% reduction in the amount of proliferative cells in arresten tumors (p<0.001) (Figure 2D–E), at least partly explaining the smaller size of these tumors. Since arresten is a potent inhibitor of angiogenesis, the amount of tumor blood vessels was determined. The blood vessel density reduced almost 50% (p<0.001) in the arresten xenografts relative to the control tumors (Figure 2F–G). Histological analysis of HSC-3 xenografts revealed that besides being smaller the Arr-HSC tumors also more often contained central keratinized areas and keratin pearls, indicating higher degree of differentiation, and the proportion of the surrounding poorly differentiated tumor cell layer was smaller than in the control tumors (Figure S6A–D). E-cadherin staining showed either diffuse cytoplasmic signals in the poorly differentiated tumor areas, or membranous staining within the keratinized areas in all xenografts (Figure S6E–H).

### Arresten Inhibits HSC-3 Carcinoma Cell Invasion in the 3D Organotypic Model

**Figure 3:**
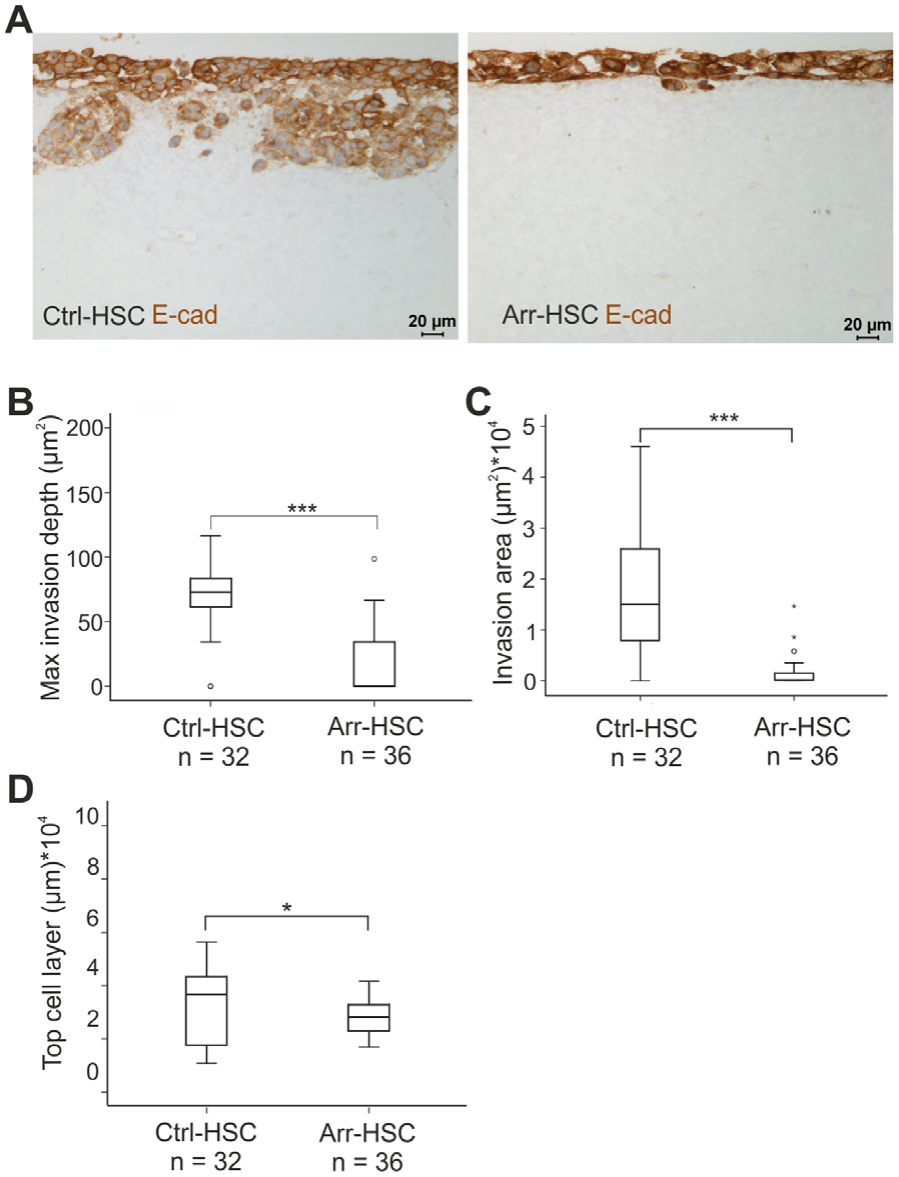
Arresten efficiently inhibits HSC-3 carcinoma cell invasion in an organotypic model. **A.** Ctrl-HSC and Arr-HSC cells (7×10^5^) were cultured on top of a collagen gel embedded with human gingival fibroblasts (7×10^5^). The organotypic sections were stained with E-cadherin antibody (brown). Scale bar 20 µm. Tumor cell invasion and growth were quantified by measuring the maximal invasion depth (**B**), invasion area (**C)** and area of the top cell layer of pancytokeratin stained sections (**D**). Mann-Whitney U-test, ***p<0.001, *p<0.05, (n = total number of fields analyzed, 4–5 fields per organotypic section).

To further explore the invasive properties of the Arr-HSC cells and to gain insight into the mechanisms of action of arresten, we performed three-dimensional (3D) organotypic assays in which HSC-3 carcinoma cells were allowed to invade into a collagen matrix supplemented with human gingival fibroblasts. After a 2-weeks culture period, the organotypic sections were immunostained with E-cadherin and pancytokeratin AE1/AE3 antibodies, and the maximal invasion depth and area, and the thickness of the top cell layer were determined. As expected, the Ctrl-HSC cells invaded deep into the collagen matrix and E-cadherin staining clearly decreased in the matrix-invaded cells indicating loosening of the cell-cell contacts during the invasion (Figure 3A–B). Arresten overexpression almost completely blocked HSC-3 cell invasion, the maximal invasion depth and the area of invading cells being significantly smaller than those of the control cells. Relative to the Ctrl-HSC cells, the Arr-HSC cells also formed a very thin top cell layer, with prominent membranous E-cadherin staining (Figure 3A–D).

### Arresten Overexpression Promotes an Epithelial Morphology and E-cadherin in Cell-cell Contacts

**Figure 4:**
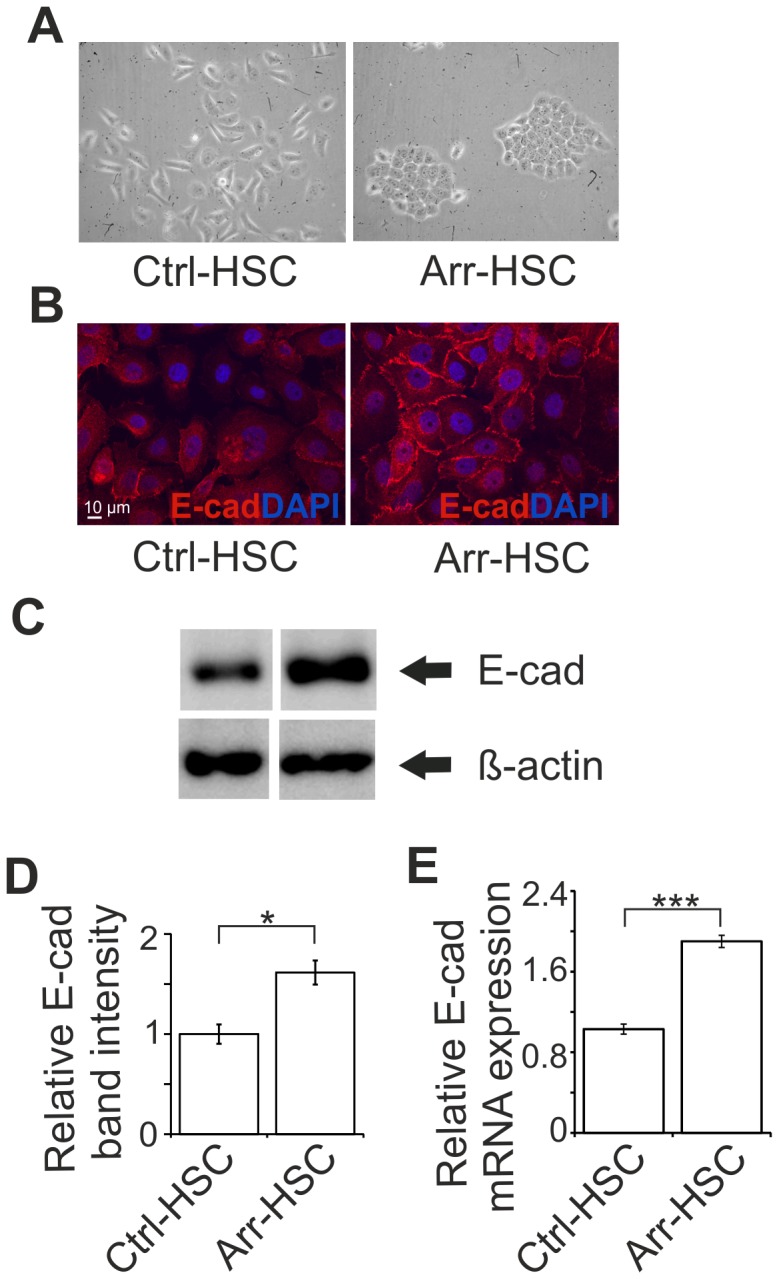
Arresten promotes an epithelial morphology of HSC-3 cells and increases the amount E-cadherin in cell-cell contacts. **A.** Arresten overexpression induced a cobblestone-like appearance in HSC-3 tongue squamous cell carcinoma cells (200×magnification). **B.** Immunostaining of E-cadherin (red) in cultured Ctrl-HSC and Arr-HSC cells (blue, DAPI). Scale bar 10 µm. **C.** 10 µg of total protein from lysed cell extracts was analyzed by Western blotting with E-cadherin antibody. β-actin was used as a loading control. **D.** The relative band intensities were quantified (n = 3 Western analyses from separate protein extractions; mean ± SEM). **E.** mRNA expression of E-cadherin in cultured Ctrl-HSC (N = 6, n = 12) and Arr-HSC (N = 3, n = 6) cells (N = number of clones analyzed; n = number of samples analyzed). The expression levels were normalized to that of the GAPDH housekeeping gene and are presented relative to values obtained for Ctrl-cells (mean ± SEM) Students t-test, ***p<0.001, *p<0.05.

Besides the non-migratory and less invasive phenotype of Arr-HSC cells observed in the previous assays, we noticed a prominent change in their cell morphology. Compared to the control HSC-3 cells, the Arr-HSC clones displayed a flatter, less spindle-shaped phenotype and they grew in aggregated cobblestone-like clusters (Figure 4A). Similar morphological changes were observed in MDA-MB-435 breast carcinoma cells in the presence of excess arresten (Figure S2E), These findings led us to hypothesize that arresten may affect the epithelial plasticity of the HSC-3 cells, and induce a switch from the mesenchymal carcinoma cell phenotype to a one resembling normal epithelial cells. The carcinoma cells undergo EMT-like events during cancer progression, and a reversed process MET is suggested to occur, endowing a less motile phenotype [22], [31]. Accordingly, we further investigated whether arresten overexpression could restore the epithelial characteristics of the tumor cells. The Arr-HSC cells growing in tightly packed clusters expressed more epithelial marker E-cadherin on their cell surfaces than the Ctrl-HSC cells (Figure 4B), which is likely to contribute to their epithelial-like morphology and reduced motility. Besides the recruitment of E-cadherin to the Arr-HSC cell membrane, its expression in these cells was increased when compared to the Ctrl-HSC cells (Table S1, Figure 4C–E). The amount of E-cadherin mRNA in the Arr-HSC cells was 1.9-fold ±0.06 (p<0.001) (Table S1, Figure 4E), and that of protein 1.6-fold ±0.12 (p = 0.019), both significantly higher than in control cells (Figure 4C–D). Strong immunofluorescence signals for the mesenchymal marker vimentin were observed in some individual Ctrl-HSC and Arr-HSC cells, but evident differences in these signals could not be detected between the cell lines (Figure S7).

### Arresten Affects Cell Proliferation and Apoptosis of HSC-3 Cells in vitro

**Figure 5:**
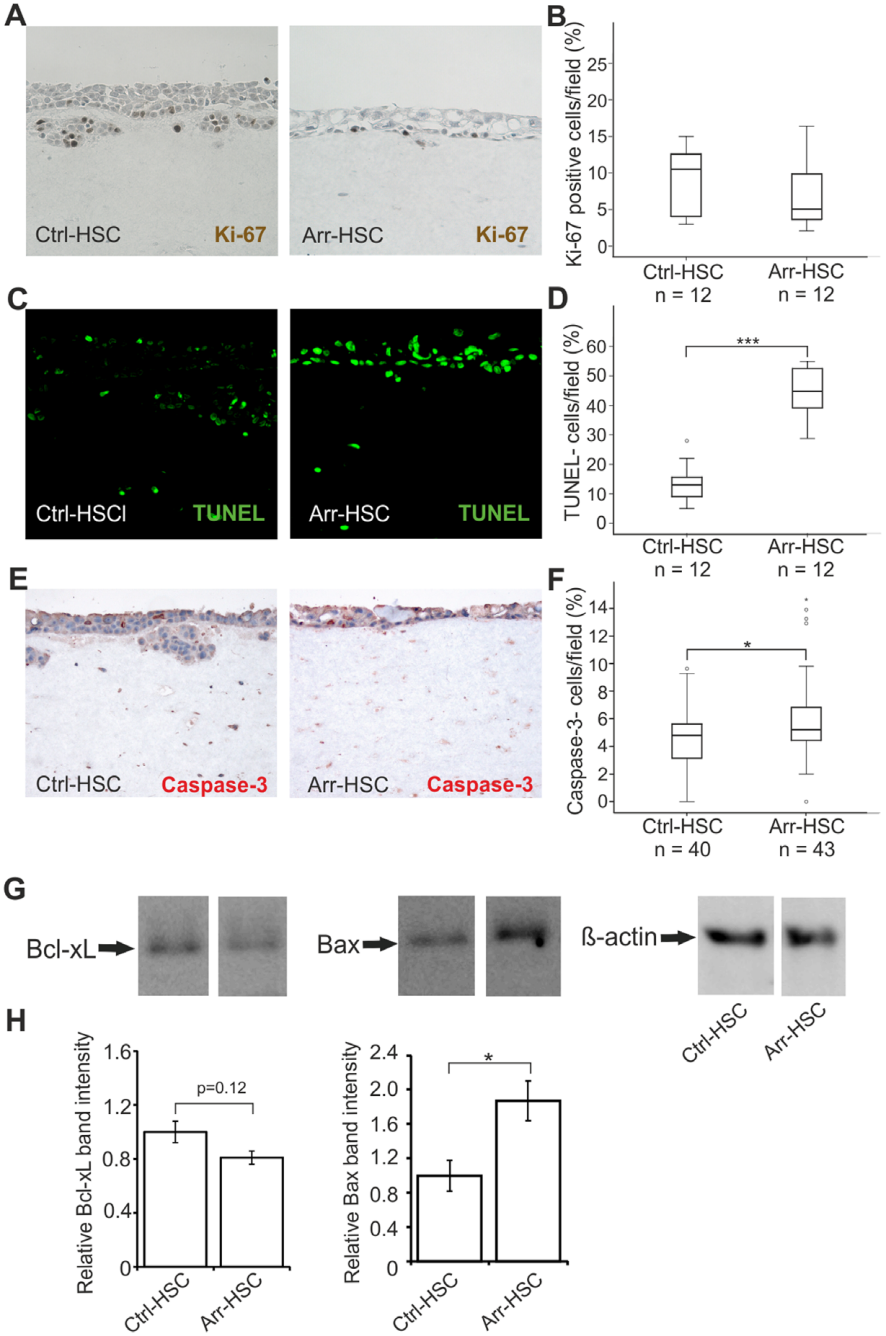
Arresten increases apoptosis of HSC-3 carcinoma cells in the organotypic model. **A.** Organotypic sections were stained for the proliferation marker Ki-67 (brown). **B.** Proliferation was defined as a percentage of Ki-67-positive cells among the total number of carcinoma cells per microscopic field (200×magnification; n = total number of fields analyzed, 3–5 fields per organotypic section). **C–F.** Apoptotic cells were detected by TUNEL assay (green) and caspase-3 staining (red). Apoptotic cell death was quantified in terms of TUNEL (**D**) and caspase-3-positive (**F**) cells as a percentage of total number of carcinoma cells per microscopic field (200×magnification; n = total number of fields analyzed, 3–5 fields per organotypic section). Mann-Whitney U-test, ***p<0.001, *p<0.05. **G.** 20 µg of total protein of lysed cell extracts was separated by SDS-PAGE and immunoblotted with antibodies against signaling molecules of the Bcl-family apoptosis pathway, anti-apoptotic Bcl-xL and pro-apoptotic Bax. β-actin was used as a loading control. **H.** The relative band intensities were quantified (n = 3 Western analyses from separate protein extractions; mean ± SEM). Students t-test, *p<0.05.

We next wished to determine the reason underlying the thin top cell layer formed by the Arr-HSC cells in the organotypic model, and set out to study tumor cell proliferation and apoptosis. The number of proliferating Ki-67-positive tumor cells was smaller, but not statistically significant, in the Arr-HSC than in the Ctrl-HSC 3D cultures (Figure 5A–B), which is in agreement with our observation on reduced tumor cell proliferation in Arr-HSC xenografts (Figure 2D–E). The TUNEL assay showed that the Arr-HSC cells underwent apoptosis more often than the control cells in the 3D model (Figure 5C–D). Since the TUNEL assay also detects other types of cell death in addition to apoptosis, we wanted to confirm our finding by caspase-3 staining. We observed a similar and significant (p = 0.030) trend on increased apoptosis in Arr-HSC cells (Figure 5E–F) although the increase was milder than the one in the TUNEL assay. In HSC-3 xenografts, however, only few TUNEL-positive cells were detected mainly in the keratinized central tumor areas (Figure S8). We have previously shown that recombinant arresten affects mitochondrial apoptosis-related Bcl-family signaling molecules in microvascular endothelial cells [18]. In the current experiment the pro-apoptotic Bax protein showed 1.9-fold ±0.23 increase in the Arr-HSC cells relative to the Ctrl-HSC cells (p = 0.046), whereas the anti-apoptotic Bcl-xL protein level concomitantly showed a decreased, although not statistically significant, trend to 0.8-fold ±0.049 of the controls (p = 0.12), thus shifting the balance towards a situation favoring apoptosis (Figure 5G–H).

### Electric Cell-substrate Impedance Sensing Reveals Alterations in Arr-HSC Cell Spreading and Cell-cell Contacts

**Figure 6:**
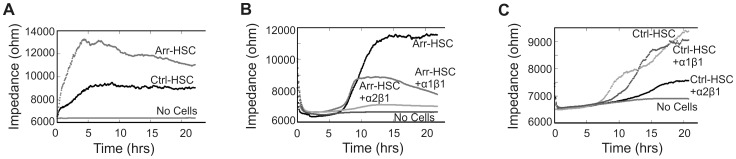
Arr-HSC cell spreading is impaired in the presence of a function-blocking antibody to α1 integrin. A. The impedance, reflecting cell adhesion and spreading, was measured for Ctrl-HSC and Arr-HSC cells using electric cell-substrate impedance sensing (ECIS) (mean of duplicate wells of representative ECIS plates). The Arr-HSC cells showed markedly higher impedance at a low frequency than the control cells. **B**. Treatment of Arr-HSC cells with a specific function-blocking α1 antibody reduced the impedance when compared to the untreated Arr-HSC cells. Treatment of Arr-HSC cells with integrin α2 antibody almost completely abolished the cell spreading. **C**. Ctrl-HSC cells showed reduced spreading in the presence of integrin α2 antibody while α1 antibody had no effect on impedance.

To pursue the mechanisms underlying the altered behavior and morphology of Arr-HSC cells we performed measurements using electric cell-substrate impedance sensing (ECIS), a method that provides quantitative data on cell attachment, spreading and the strength of cell-cell contacts by monitoring changes in the system impedance [35]. The Arr-HSC cells showed markedly higher impedance at a low frequency than the control cells (Figure 6A). Also the HSC-3 cells treated with ArrCM showed higher impedance than those treated with CtrlCM (Figure S9A). The change in the impedance can be related either to cell inherent dielectric properties, formation of cell-cell junctions or cell-substrate interactions, and a mathematical ECIS™ Model can be applied to distinguish these parameters from each other [36]. Thus, a cell membrane capacitance (Cm) reflects the structure and folding of cell membrane, a barrier resistance (Rb) refers to establishment of cell-to-cell junctions, and a cell-substrate interaction parameter α is linearly related to the cell surface area and, inversely, to the distance between cell and substrate [36]–[38]. This modeling supported our observations on altered cell morphology and E-cadherin of the Arr-HSC cells. First, significantly increased Rb of the Arr-HSC cells relative to the Ctrl-HSC implied tightening of intercellular junctions (Figure S9C). As we did not observe apparent changes in the cell size between the HSC clones by phase contrast microscopy or immunofluorescent stainings (Figure 4A–B), the higher α value of Arr-HSC cells can be attributed to a better cell adhesion to the substratum (Figure S9D). Lastly, altered Cm of Arr-HSC cells further points to differences in the cell morphology and cell membrane properties (Figure S9E).

The signaling through β1 integrin is known to affect E-cadherin dynamics, and cell motility and EMT are abrogated by integrin knockdown [38]–[41]. α1β1 integrin was identified as a functional receptor for arresten on endothelial cells [3], [16], [18], but to date the arresten receptors on carcinoma cells have not been identified. HSC-3 cells express several integrin receptors, including α1β1 and α2β1 (unpublished FACS data). We thus performed ECIS experiments with Arr-HSC cells in the presence of function-blocking antibodies for collagen binding integrins α1β1 and α2β1. Administration of integrin α1 antibody decreased the impedance of the Arr-HSC cells while that of the control cells remained unaltered (Figure 6B–C). Incubation of Arr-HSC cells with the integrin α2 blocking antibody almost completely inhibited the cell spreading, but also control cells showed reduced impedance in the presence of this antibody. Control IgG did not have any effect on the behavior of the cells (Figure S9B). These data suggest that integrin α1β1 is able to bind arresten also on oral squamous carcinoma cells, resulting in changes in the cell morphology and motility.

## Discussion

Tumor growth and metastasis depends on local neovascularization induced by hypoxic conditions and regulated by the tumor microenvironment, including the components of the ECM. Arresten is one of the five thus far identified basement membrane collagen IV-chain-derived fragments that can inhibit angiogenesis and thereby reduce tumor growth via integrin binding [3]–[13], [15]. Arresten binds to integrin α1β1 on endothelial cells to regulate the actin cytoskeleton and migration [3], [16], [18]. Besides the expected anti-angiogenic effect of arresten in mouse xenograft tumors, we demonstrate here that it directly affects oral carcinoma cells both *in vivo* and *in vitro*. This is the first time that the direct effects of arresten on other cell types than endothelial cells have been studied in more detail.

Here the overexpression of arresten strongly inhibited oral squamous cell carcinoma cell invasion in Matrigel Transwell assay and in organotypic 3D model. Arresten also clearly reduced the migration of these cells, as well as MDA-MB-435 carcinoma cells, in monolayer culture. In an *in vivo* tumor burden model arresten overexpression led to a smaller tumor size, impaired angiogenesis, and changes in tumor tissue architecture. Since human subcutaneous xenograft tumors rarely metastasize in nude mice [42], we assessed the amount of local invasion and found that Arr-HSC tumors invaded less into the surrounding tissue than the control tumors.

In order to explore the reasons underlying the significantly smaller size of subcutaneous Arr-HSC xenografts and thin top cell layer formed by the Arr-HSC cells in the organotypic model, we analysed tumor cell proliferation and apoptosis in these samples. Compared to Ctrl-HSC cells, a reduced number of proliferating Ki-67-positive Arr-HSC cells were detected in both models. Furthermore, the MTT assay showed a smaller number of viable HSC-3 cells in response to arresten in long-term monolayer culture, although previously we did not observe increased apoptosis-related caspase-3 activity of HSC-3 cells by short-term exposure to recombinant arresten [18]. Arresten has been shown to exert a pro-apoptotic effect on various types of endothelial cells *in vitro*, and both on endothelial and tumor cells in an *in vivo* mouse tumor burden model [16], [18]. Our current findings show significantly increased number of TUNEL-positive cells and also a slightly elevated number of caspase-3 positive cells in the 3D organotypic model involving Arr-HSC cells by comparison with Ctrl-HSC cells. Bcl signaling is affected by arresten in both endothelial cells and, according to our current data, also in carcinoma cells (Figure 5G–H and [18], [19]); the expression of anti-apoptotic Bcl-xL decreased in both cell types, but the amount of pro-apoptotic Bax increased only in the Arr-HSC carcinoma cells. Nevertheless, the net result in both cell types is a shift in the balance of pro-apoptotic and anti-apoptotic stimuli in a direction that favors apoptosis. In subcutaneous xenografts, however, only few apoptotic cells were detected that were located mainly in dyskeratotic areas. It seems to depend on the composition of the surroundings whether the cells are responding to arresten by reduced proliferation or increased apoptosis. However, in the end the net result in both experimental set-ups is the same: smaller xenografts in mice and thin top cell layer in 3D model. Taken together, we consider likely that besides inducing apoptosis arresten can also reduce the proliferation of HSC-3 cells, which leads to reduced tumor growth via two routes.

Another clear effect that arresten overexpression had on carcinoma cells was the change in their morphology. Both the Arr-HSC and Arr-MDA cells grew in aggregates that were tightly attached to each other, whereas the control cells displayed a more spindle-shaped and mesenchymal-like morphology (Figure 4A, Figure S2E). This was concomitant with up-regulation of E-cadherin expression and its localization in cell-cell contacts in the Arr-HSC cells. Histopathologic evaluation of subcutaneous xenografts suggested that arresten overexpression affected tumor differentiation *in vivo*, Arr-HSC tumors containing more often keratinized areas and keratin pearls than Ctrl-HSC tumors. The clear membranous E-cadherin staining was localized around these keratinized areas. The ECIS experiments and modeling (Figure 6A, Figure S9) also supported our notion that HSC-3 cells form tighter cell-cell and cell-substrate contacts in the presence of arresten. The loss or down-regulation of cell-cell adhesion is crucial for the cells to metastasize, and it is considered to be one of the key features of EMT [43]. EMT-like changes are reversible, however, and thus the cells can restore their non-motile epithelial characteristics in the MET process. Approximately a 2-fold excess of E-cadherin in A431 human epidermoid carcinoma cells has been shown inhibit their invasion [41] which is in line with the degree of E-cadherin up-regulation induced by arresten in our experiments (Table S1). Our data therefore suggest that carcinoma cells undergo changes resembling MET in the presence of arresten.

Arresten mediates its effects on endothelial cells through integrin receptors. Arresten is known to bind to α1β1 integrin and this ligation is shown to lead to the inhibition of focal adhesion kinase (FAK)/c-Raf/MEK1/2/p38/ERK1 mitogen-activated protein kinase pathway and suppression of endothelial cell migration, proliferation, and tube formation [16]–[19], [44]. Integrin α1 is also required for the anti-survival effect of arresten in endothelial cells [18]. Using ECIS measurements we showed here that the high impedance of Arr-HSC cells was reduced upon treatment with the function-blocking α1 integrin antibody. These data suggest that α1β1 integrin mediates the promoting effect of arresten on HSC-3 cell-cell contacts and cell spreading that are disturbed upon antibody binding. Blocking of α2β1 integrin receptor had a strong inhibitory effect on both the Arr-HSC and the Ctrl-HSC cell attachment suggesting that this receptor mediates interactions that do not involve arresten. Thus, the upregulation of E-cadherin on cell-cell junctions and the concomitant less invasive behavior may be linked to modulation of integrin α1β1 signalling by arresten. The manipulation of β1 integrin and subsequent signaling pathways can lead to reversion of the malignant phenotype [45]–[46]. The ECM proteoglycan versican, known to interact and signal through β1-integrin [47], was recently shown to induce MET in MDA-MB-231 cells [48] further supporting the concept that alterations in the ECM can regulate epithelial plasticity. We also consider it possible that the excess of arresten disturbs the cell-matrix interactions in the collagen I-based 3D organotypic model resulting in induction of cell death.

ECM molecules, such as collagen I, for example, induce EMT by an integrin and FAK-mediated regulation of cadherins, both by disrupting E-cadherin adhesion complex and by upregulating N-cadherin expression [39], [27], [49]. A correctly assembled collagen IV network supports the differentiated epithelial cell phenotype, and disruption of this network by administration of the α1(IV)NC1 domain has been shown to facilitate EMT in mouse proximal tubular epithelial cells *in vitro* [50]. This observation differs from the epithelial morphology-promoting effect of arresten on oral carcinoma cells shown here, but these two phenomena represent distinct types of transitions [22] and diverse cells may respond in a different manner to stromal signals. Assadian *et al*. published recently a study which shows that p53 can induce an anti-angiogenic program whereby expression of α1(IV) chain is upregulated, stabilized by prolyl-4-hydroxylase and efficiently processed by MMPs to an arresten-containing peptide [20]. This p53-dependent ECM remodeling was suggested to destabilize the vascular collagen IV network and thereby prevent endothelial cell adhesion and migration leading to reduced angiogenesis and tumor growth *in vivo* and *in vitro*. Our observations on the inhibition of tumor angiogenesis and growth by arresten are in line with these observations, but our data suggest that arresten also reduces proliferation, induces apoptosis and facilitates epithelial plasticity in tumor cells. As tumor cells respond to many biologically active molecules in biphasic manner [51]–[52], the effects of arresten may also vary depending on its level. To date, the systemic or local concentration of arresten is not known [20], although a pilot study by Ramazani et al. suggests that the normal circulatory level of collagen IV is around 100 ng/ml in healthy humans giving us some cues on the level or arresten [53].

We show here for the first time that arresten directly modulates the behavior of carcinoma cells, and propose that this occurs at least partially via binding to integrin α1β1. Oral squamous cell carcinoma and breast carcinoma cells overexpressing arresten changed to a more epithelial-like phenotype, possibly reflecting ongoing MET-like events, and subsequently became less motile and more apoptotic. However, the MET-like events may not always be beneficial for survival, as MET has also been reported during the establishment of metastases. Furthermore, some ECM molecules have been found to contribute to the formation of premetastatic niches [30], [54]. In summary, since arresten is a potent inhibitor of angiogenesis, and also exerts strong anti-invasive effects on carcinoma cells, it could be considered a candidate for drug development efforts. However, the MET-inducing property of arresten and its role in primary tumors and metastases should be first characterized in detail.

## Materials and Methods

### Ethics Statement

The tumor experimentation in mice was approved by the ethics committees of the State Provincial Offices of Oulu and Southern Finland (permit numbers OLH-2006-02521/Ym-23, OLH-2006-01987/Ym-23, ESLH-2008-03956/Ym-23, ESLH-2008-09631/Ym-23). The carcinoma cell injections were performed under isofluran anesthesia, and every effort was made to minimize suffering, e.g. by using Rimadyl for pain relief after injections. A tumor diameter of more than 10 mm was the criterion for euthanasia.

### Cell Culture

The culturing of the HSC-3 human tongue squamous carcinoma cells (JCRB) and primary human fibroblasts obtained from biopsies of healthy gingiva [55] is described in the supplemental methods (Text S1).

### Plasmid Constructs Transfection and Selection of HSC-3 and MDA-MB-435 Cells Expressing Arresten

The cDNA coding for human arresten construct [18] (a kind gift from Raghu Kalluri, Beth Israel Deaconess Medical Center, Harvard Medical School, Boston, MA) was cloned into the pcDNA3.1 expression vector (Invitrogen). HSC-3 and MDA-MB-435 cells were transfected with these plasmids, or with an empty pcDNA3.1 vector. The transfected cells were selected with Geneticin G418 antibiotic (Invitrogen) to obtain stable populations of cells expressing human arresten. The clones used in the experiments were named Ctrl-HSC, Arr-HSC(1), Arr-HSC(2), Ctrl-MDA, Arr-MDA(1) and Arr-MDA(2). Detailed cloning and transfection protocols are presented in supplemental methods (Text S1).

### Purification of Recombinant Arresten

Recombinant arresten was purified from culture media of HEK- 293 cells that had been stably transfected with the arresten plasmid described above. Recombinant arresten was purified from the conditioned media using an ANTI-FLAG^R^ M2 affinity column (Sigma-Aldrich). Details are described in supplemental methods (Text S1).

### Transwell Migration Assays

The Transwell migratory capacities of the Ctrl-HSC and Arr-HSC cell lines were studied by plating cells into 6.5-mm diameter and 8.0-µm pore size membrane Transwell inserts (Costar). The inserts were equilibrated in serum-containing medium for 2 h, the cells were trypsinized and 30 000 cells in 100 µl of serum-containing medium were plated into each well and 600 µl of serum-containing medium was added to the lower chamber. Alternatively, native HSC-3 cells were suspended into media with purified recombinant arresten (0, 5, or 20 µg/ml), and same amount was also added to the lower chambers. The cells were allowed to migrate overnight, fixed in 10% TCA, washed and stained with 0.1% crystal violet. The cells that had migrated to the underside of the membrane were counted under a Leica DMRB microscope (Leica Microsystems). In the co-culture migration assays the inserts were equilibrated for 2 h with media collected from Arr-HSC cells for 24 h. The Ctrl-HSC cells were then suspended in the co-culture medium and plated on the Transwell wells as described above.

### Scratch Wound Healing Assay

Cells were seeded on chamber slides (Lab-Tek, Nunc) and grown to confluence. A cell-free wound was generated by scraping the confluent monolayer with a pipette tip (500 µl, ART, Molecular Bioproducts). The cells were fixed, stained with crystal violet, examined under a microscope (Leica Microsystems) and photographed at 0, 16 and 48 h time points. The width of the wound in each high power field (50×) was quantified (2–3 wounds were generated per chamber, at least three pictures were taken of each wound and at least three measurements were performed on each picture).

### Tumor Xenografts in Nude Mice

One million Ctrl-HSC and Arr-HSC cells in 200 µl of serum-free media were subcutaneously injected into both flanks of 11-week-old Balb/c nu/nu nude female mice (Harlan). Each group contained ten mice. Tumor growth was measured at days 6, 13 and 16, and tumor volumes were calculated with the formula length×width^2^×0.52. At day 16, the mice were sacrificed and the tumors were collected for histology.

### Histology and Immunohistochemistry of the Xenografts

The tumors were fixed in 4% neutral buffered formalin overnight. 5-µm sections were deparaffinized and stained with Mayeŕs hematoxylin-eosin. A pathologist evaluated the degree of invasion of each hematoxylin-eosin-stained tumor in a blinded fashion. To detect the numbers of proliferating cells in HSC-3 xenografts, tumor sections were stained with Ki-67-antibody (Dako) as described previously [56]. Quantification was performed by counting the number of Ki-67 positive cells relative to non-stained cells in each high power field (400×magnification). Blood vessels were stained with CD31 (BD Biosciences PharMingen), according to a previously published protocol [57], and their numbers were counted.

### Immunofluorescent Staining of Cells

Cells grown on coverglasses were fixed for 10 min in 4% paraformaldehyde–PBS, blocked and permeabilized for 20 min with 0.5% BSA/0.2% gelatine/0.1% Triton X-100 in PBS and incubated with primary anti-E-cadherin (Cell Signaling Technology) and anti-vimentin Ab-2 (NeoMarkers) antibodies in 0.5% BSA/0.2% gelatine–PBS overnight at 4°C. The secondary Cy2 and Cy3-conjugated antibodies (Jackson ImmunoResearch Laboratories) were applied for 45 min at room temperature. 4′,6′-diamino-2-phenylindole hydrochloride (DAPI) was added to visualize the cell nuclei. Confocal images were captured using a laser confocal microscope (Olympus IX81).

### Organotypic Cultures

In the organotypic assays the carcinoma cells were allowed to invade into a 3-D mixture of collagen and human gingival fibroblasts. The collagen gels were prepared as previously described [56]. Briefly, 8 volumes of collagen type I (3.45 mg/ml; BD Biosciences), 1 volume of 10×DMEM (Sigma) and 1 volume of FBS with gingival fibroblasts (7×10^5^ cells) were allowed to polymerize at 37°C for 30 min. After polymerization, 7×10^5^ HSC-3 cells (Ctrl-HSC and Arr-HSC) were added to each gel. The gels were lifted onto collagen-coated (BD Biosciences) nylon discs (Prinsal Oy) resting on curved steel grids that were placed on 6-well plates. Medium was added to reach the undersurface of the grid, generating an air-liquid interface. Quantification of invasion was performed to identify carcinoma cells according to a previously published protocol based on pancytokeratin immunostaining (AE1/AE3 antibody, Dako) [56]. Briefly, the areas of immunostained non-invading and invading cells were calculated, and the average invasion depth per microscopic field (the distance of the invaded cell clusters from the lower surface of the non-invasive cell layer) was measured in each sample according to a previously published protocol [56].

### Histology and Immunohistochemistry of the Organotypic Cultures

The organotypic discs were fixed in 4% neutral buffered formalin overnight. 6-µm sections were deparaffinized and stained with Mayeŕs hematoxylin-eosin. For immunohistochemistry of pancytokeratin AE1/AE3 antibody (Dako) the endogenous peroxidase activity was blocked with 0.3% H_2_O_2_ in MeOH for 30 min. Antigen retrieval was performed with 0.4% pepsin in 0.01 M HCl at 37°C for 1 h or by microwaving (T/T Mega) the sections in citrate buffer (REAL Target Retrieval Solution, pH 6; Dako) or in Tris/EDTA (10 mM Tris, 1 mM EDTA, pH 9) for 20 min. The sections were blocked with normal serum (Vector Laboratories) in 2% BSA/PBS for 30 min and incubated with primary antibody at 37°C for 30 min and at 4°C overnight. Biotinylated secondary antibody (Vector) was applied for 1 h and StreptABComplex/HRP (Dako) in 0.5 M NaCl/PBS for 30 min. E-cadherin (Cell Signaling Technology) was stained using the REAL EnVision Detection System (Dako) according to the manufactureŕs instructions; it was incubated at 4°C overnight, after which the secondary antibody was applied for 30 min. The presence of the antigen was visualized using DAB Peroxidase Substrate (Vector) for 3 min and counterstained. In the negative controls normal serum or IgG of the appropriate species (Dako) was used instead of the primary antibody. Organotypic cultures were stained with Ki-67 (Dako) to detect the numbers of proliferating cells as described previously [56]. Quantification was performed by counting the number of Ki-67 positive cells relative to non-stained cells in each high power field (200×magnification).

### Apoptosis Assays

The terminal deoxynucleotidyl transferase-mediated dUTP nick end-labelling (TUNEL) assay and caspase-3 immunostainings were used to quantify the apotosis of cells overexpressing arresten and control HSC-3 cells grown in organotypic cultures. In the TUNEL assay the apoptotic cells were labeled according to the instructions of the *In Situ* Cell Death Detection Kit (Roche). The bright green apoptotic nuclei were viewed with a DMRB photo microscope connected to a DFC-480 camera using QWin V3 software (Leica Microsystems). Quantification was performed by counting the TUNEL positive green cells relative to the non-stained cells in each high power field (200×). For the caspase-3 staining the antigen retrieval was done by boiling in 0.01 M EDTA. The staining was done with Histomouse SP-KIT (Invitrogen). The primary antibody (cleaved caspase-3 D175, 1∶200 in 1% BSA in PBS, R&D Systems) was incubated overnight at +4°C. The slides were incubated in secondary antibody for 30 min RT and the enzyme conjugate for 10 min. Subsequently, the slides were incubated in substrate-chromophore mixture for 8 min RT, and embedded in Cole hematoxylene and mounted.

### ECIS Assays

Electric cell-substrate impedance sensing (ECIS) (Applied Biophysics Inc) was used to study cell adhesion. The cells were trypsinized and 400 000 cells in 400 µl were seeded on an 8-well ECIS plate to monitor cell spreading by means of impedance. Before plating the cells were treated 10 µg/ml integrin α1 or α2 blocking antibodies (mouse anti-human clone FB12, Millipore or goat anti-human N-19, Santa Cruz, respectively) or control IgG (Dako) for 15 min on ice. A mathematical ECIS™ model of the impedance changes was used to refine the ECIS data and to calculate cell morphological parameters (the barrier function of the cell layer, Rb; the spacing between the cell and the substratum, α; and the cell membrane capacitance, Cm [36]–[37].

### Statistical Analysis

SPSS 16.0 software was used for the statistical calculations. The results are presented as medians [25^th^ percentile, 75^th^ percentile] in boxplots, where the whiskers represent data points lying within 1.5 interquartile ranges of the median. The qPCR data, scratch wound healing results, quantification of Western blot band intensities and tumor growth curves are presented as means +/− standard error of mean (SEM). The Mann-Whitney U-test or Students t-test were used to determine statistical significance.

## Supporting Information

Figure S1 **Characterization of stable HSC-3 and MDA-MB-435 cell clones overexpressing arresten by Western blotting.** The secretion of recombinant arresten in stably transfected HSC-3 and MDA-MB-435 cells was verified by Western blotting. The cell culture medium was concentrated by acetone precipitation and the proteins were separated by SDS-PAGE and immunoblotted with anti-Flag antibody. Representative immunoblots of HSC-3 vector control Ctrl-HSC(1) and the HSC-3 arresten clones Arr-HSC(1) and Arr-HSC(2) **(A)**, and MDA-MB-435 vector control Ctrl-MDA(1) and theMDA-MB-435 arresten clones Arr-MDA(1) and Arr-MDA(2) (**B**).
(TIF)

Figure S2 **Arresten inhibits migration of HSC-3 and MDA-MB-435 cells, and induces morphological changes in MDA-MB-435 cells** ***in vitro*** **.** **A.** Wound closure in a scratch wound healing assay was markedly slower in both Arr-HSC clones than in the Ctrl-HSC cells. Scale bar 50 µm. **B**. MDA-MB-435 wounds did not close within 48 h, but both Arr-MDA clones showed reduced migration relative to the Ctrl-MDA cells. Scale bar 50 µm. **C**. Quantification of wound closure in the Ctrl-HSC and Arr-HSC clones (n = 9 fields per clone at 0 h, n = 18 at 16 and 48 h). Mann-Whitney U-test, ***p<0.001. **D.** Quantification of wound closure in the Ctrl-MDA and Arr-MDA clones (n = 26 fields per clone at 0, 16 and 48 h). Mann-Whitney U-test, ***p<0.001. **E.** Arresten overexpression induced a cobblestone-like appearance in a representative clone of MDA-MB-435 breast carcinoma cells (100×magnification).
(TIF)

Figure S3 **Overexpression of arresten reduces the viability of HSC-3 cells in 2D culture. A.** Proliferative cells were detected by BrdU labeling. 5000 Ctrl-HSC and Arr-HSC cells were allowed to attach and the cell proliferation was measured after 24 h using the colorimetric cell proliferation ELISA BrdU assay at 450 nm (n = 10 wells). **B.** Viable cells were detected by MTT assay. 5000 Ctrl-HSC and Arr-HSC cells were allowed to grow on 96-well plates for 68 hours before exposure to MTT reagent. Formed crystals inside the viable cells were dissolved in DMSO and the absorbance was measured at 540 nm (n = 18 wells). Mann-Whitney U-test, ***p<0.001.
(TIF)

Figure S4 **Conditioned Arr-HSC culture medium inhibits HSC-3 cell migration in co-culture experiments.** Conditioned media from Ctrl-HSC (CtrlCM) and Arr-HSC (ArrCM) clones were collected at 24 h and administered to Ctrl-HSC cells. **A**. Cell migration was assayed with a Transwell assay in which 30 000 cells were allowed to migrate through Transwell inserts and the number doing so was counted under a microscope with 50×magnification. Mann-Whitney U-test, ***p<0.001, (n = total number of fields analyzed, 3–5 fields per Transwell insert). **B.** Recombinant arresten is stable in co-culture at 37°C and in storage at 4°C. The CM was collected from Arr-HSC cells after 48 h culture period. ArrCM was administered to Ctrl-HSC cells and medium samples were collected after 24 h and 72 h incubations at 37°C. A sample of ArrCM stored for72 h at 4°C was also included in the analysis. The CM proteins were concentrated with acetone precipitation and analyzed by Western blotting with an anti-Flag antibody.
(TIF)

Figure S5 **Arresten inhibits cell invasion** ***in vitro*** **.** 30 000 Arr-HSC and Ctrl-HSC cells were allowed to invade through the Matrigel-coated Transwell inserts for 22 hours. The invaded cells were stained with hematoxylin and counted under a microscope with 20×magnification. Mann-Whitney U-test, *p<0.05, (n = total number of fields analyzed, 3–5 fields per Transwell insert).
(TIF)

Figure S6 **Arresten alters the tissue architecture of HSC-3 xenografts.** One million Ctrl-HSC and Arr-HSC cells were injected subcutaneously into the flanks of nude mice (n = 10 per group) and tumor growth was monitored over 16 days. Representative hematoxylin-eosin and E-cadherin stainings of HSC-3 xenografts. **A–B**. Ctrl-HSC xenografts show an appearance of poorly differentiated squamous cell carcinomas (**A**). Some tumors contain also keratinized areas (dotted line) in central tumor area (**B)**. **C–D**. Arr-HSC xenografts resemble moderately or well differentiated squamous cell carcinomas, and relative to the Ctrl-HSC tumors, show more pronounced keratinized areas (dotted line) and keratin pearls (arrowhead), sometimes surrounded by a thin layer of poorly differentiated cells (**D**). Scale bar 500 µm (A, B, D) and 100 µm (C). **E–H**. Immonostaining for E-cadherin (brown) showed either diffuse cytoplasmic signals in the poorly differentiated tumor areas (arrow), or membranous staining (open arrow) within the keratinized areas in all xenografts. Scale bar 100 µm. T = tumor; K = keratinized area; N = necrosis.
(TIF)

Figure S7 **No differences in vimentin staining between Arr-HSC and Ctrl-HSC cells.** Immunostaining of vimentin (green) in cultured Ctrl-HSC and Arr-HSC cells (blue, DAPI). Scale bar 100 µm.
(TIF)

Figure S8 **Tunel-positive cells were detected in keratinized or necrotic areas in HSC-3 xenografts.** Apoptotic cells were detected by TUNEL assay (green) in HSC-3 xenografts (blue, DAPI). Scale bar 100 µm.
(TIF)

Figure S9 **Cell-cell and cell-substrate interactions, and cell membrane capacitance show changes between Arr-HSC and Ctrl-HSC cells.** **A.** The impedance, reflecting cell adhesion and spreading, was measured for Ctrl-HSC cells treated with ArrCM or CtrlCM using electric cell-substrate impedance sensing (ECIS) (mean of duplicate wells of representative ECIS plates). HSC-3 cells treated with ArrCM showed higher impedance than those treated with CtrlCM. **B**. Ctrl-HSC cells showed reduced spreading in the presence of integrin α2 antibody while control IgG and α1 antibody had no effect on impedance. **C–E.** A mathematical ECIS™ model of the impedance changes was used to refine the ECIS data and to calculate cell morphological parameters. The barrier function of the cell layer, Rb **(C)**, and the spacing between the cell and the substratum, α (**D**), were significantly higher in Arr-HSC than in Ctrl-HSC cells. Mann-Whitney U-test, **p<0.01, *p<0.05. **E.** The cell membrane capacitance, Cm, was significantly decreased in Arr-HSC cells in comparison with Ctrl-HSC cells. Mann-Whitney U-test, **p<0.01. (n = number of ECIS wells).
(TIF)

Table S1 **Relative mRNA expression of arresten and E-cadherin in the HSC-3 and MDA-MB-435 clones.**
(DOC)

Text S1 **Supplemental methods.** Cell culture, cloning of arresten into pcDNA3.1 eukaryotic expression vector, transfection and selection of HSC-3 and MDA-MB-435 cells expressing arresten, purification of recombinant arresten, quantitative real-time RT-PCR (qPCR), cell proliferation assay, MTT viability assay, Transwell invasion assay, E-cadherin staining and apoptosis assay for HSC-3 xenografts and Western blotting.
(DOC)
