# Supplementary figures and images for: Arresten, a Collagen-Derived Angiogenesis Inhibitor, Suppresses Invasion of Squamous Cell Carcinoma
Source: PLoS One. 2012 Dec 5;7(12):e51044. doi: 10.1371/journal.pone.0051044 (PMC3515547; doi:10.1371/journal.pone.0051044)

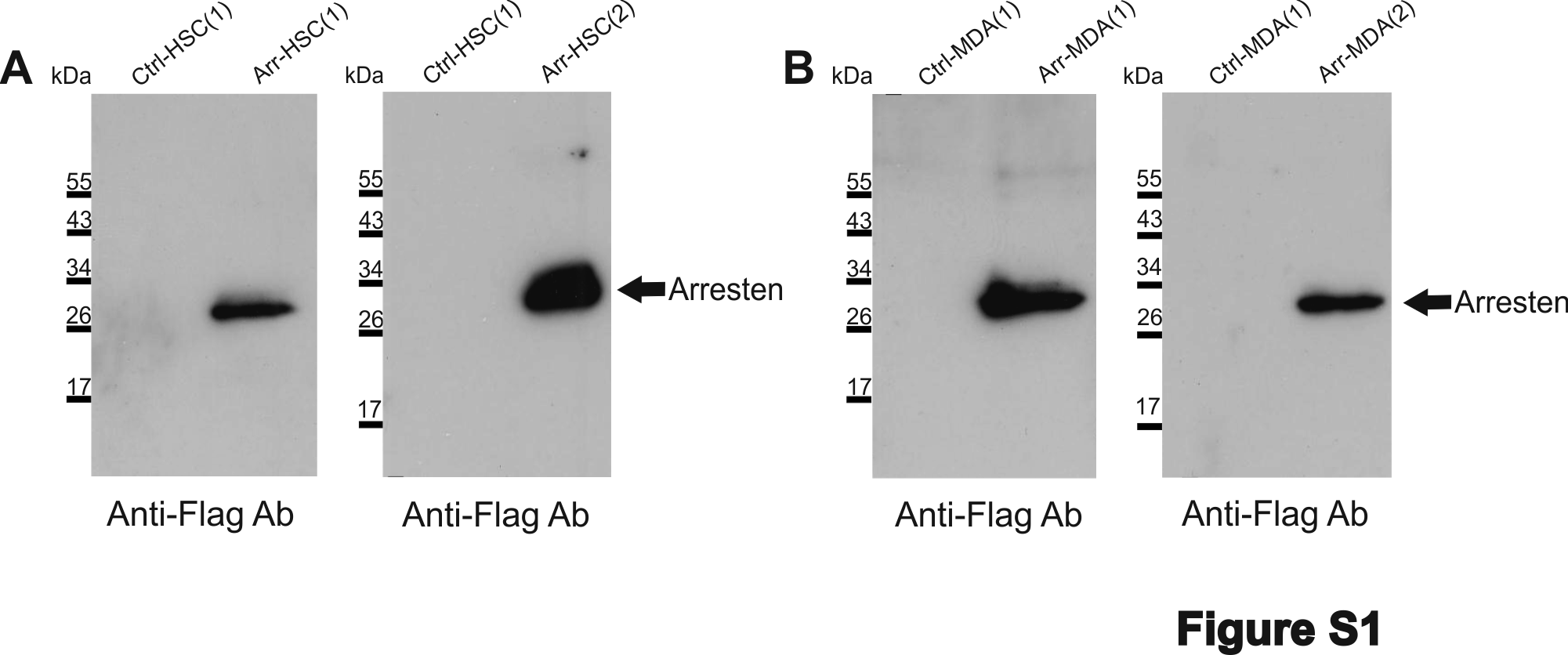

Supplement: Figure S1 — Characterization of stable HSC-3 and MDA-MB-435 cell clones overexpressing arresten by Western blotting. The secretion of recombinant arresten in stably transfected HSC-3 and MDA-MB-435 cells was verified by Western blotting. The cell culture medium was concentrated by acetone precipitation and the proteins were separated by SDS-PAGE and immunoblotted with anti-Flag antibody. Representative immunoblots of HSC-3 vector control Ctrl-HSC(1) and the HSC-3 arresten clones Arr-HSC(1) and Arr-HSC(2) (A), and MDA-MB-435 vector control Ctrl-MDA(1) and theMDA-MB-435 arresten clones Arr-MDA(1) and Arr-MDA(2) (B). (TIF) [file pone.0051044.s001.tif]

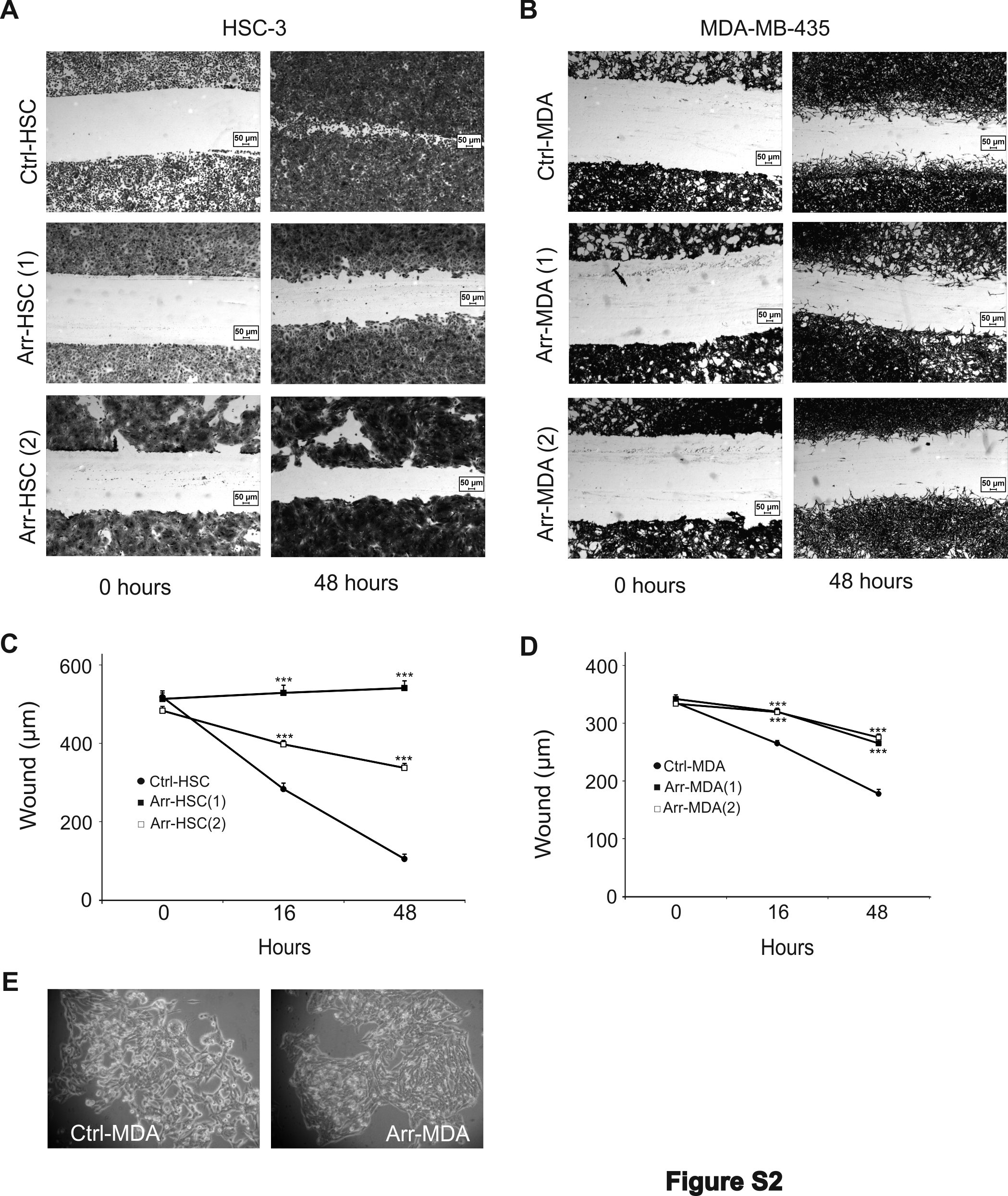

Supplement: Figure S2 — Arresten inhibits migration of HSC-3 and MDA-MB-435 cells, and induces morphological changes in MDA-MB-435 cells in vitro . A. Wound closure in a scratch wound healing assay was markedly slower in both Arr-HSC clones than in the Ctrl-HSC cells. Scale bar 50 µm. B. MDA-MB-435 wounds did not close within 48 h, but both Arr-MDA clones showed reduced migration relative to the Ctrl-MDA cells. Scale bar 50 µm. C. Quantification of wound closure in the Ctrl-HSC and Arr-HSC clones (n = 9 fields per clone at 0 h, n = 18 at 16 and 48 h). Mann-Whitney U-test, ***p<0.001. D. Quantification of wound closure in the Ctrl-MDA and Arr-MDA clones (n = 26 fields per clone at 0, 16 and 48 h). Mann-Whitney U-test, ***p<0.001. E. Arresten overexpression induced a cobblestone-like appearance in a representative clone of MDA-MB-435 breast carcinoma cells (100×magnification). (TIF) [file pone.0051044.s002.tif]

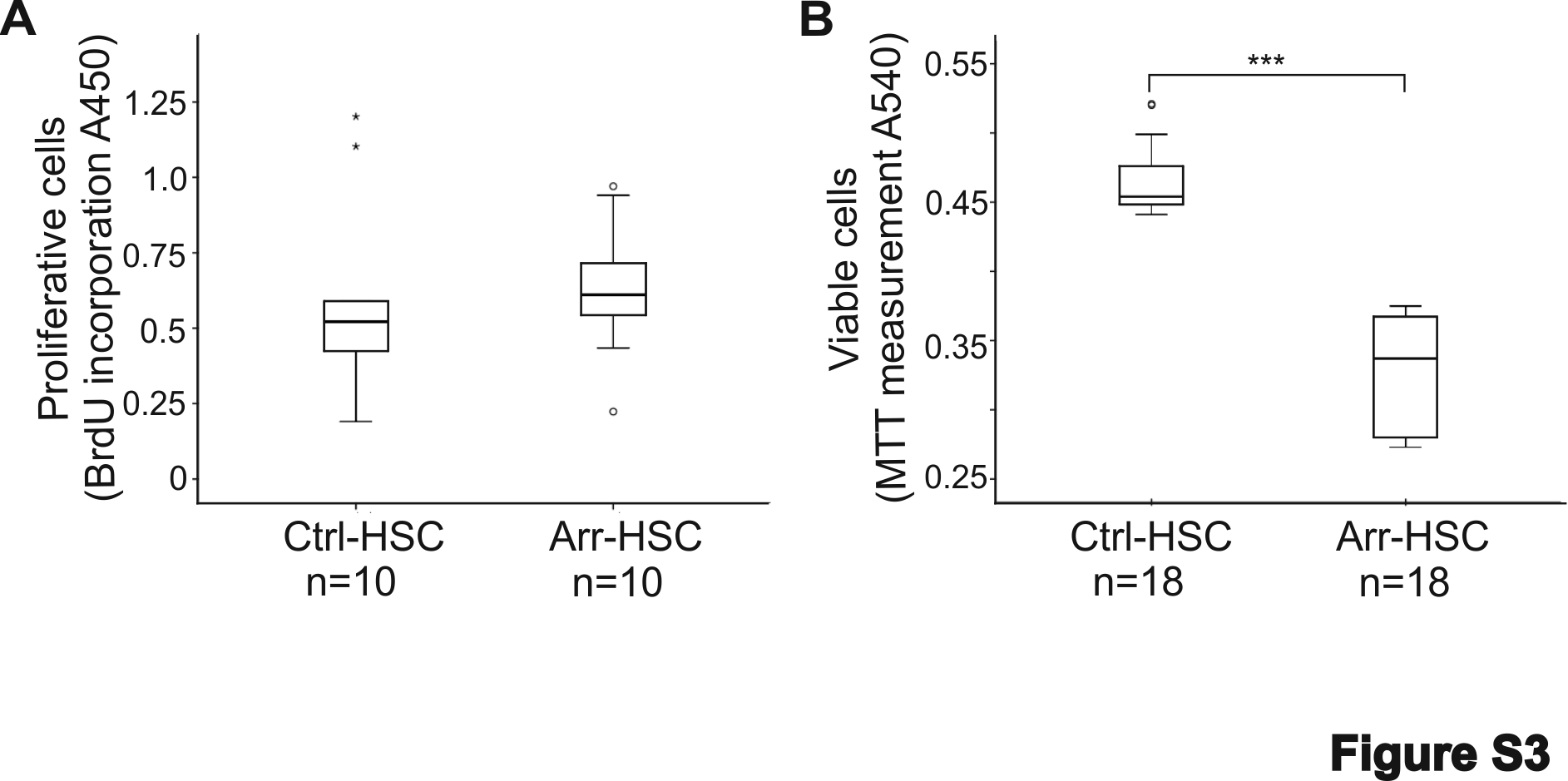

Supplement: Figure S3 — Overexpression of arresten reduces the viability of HSC-3 cells in 2D culture. A. Proliferative cells were detected by BrdU labeling. 5000 Ctrl-HSC and Arr-HSC cells were allowed to attach and the cell proliferation was measured after 24 h using the colorimetric cell proliferation ELISA BrdU assay at 450 nm (n = 10 wells). B. Viable cells were detected by MTT assay. 5000 Ctrl-HSC and Arr-HSC cells were allowed to grow on 96-well plates for 68 hours before exposure to MTT reagent. Formed crystals inside the viable cells were dissolved in DMSO and the absorbance was measured at 540 nm (n = 18 wells). Mann-Whitney U-test, ***p<0.001. (TIF) [file pone.0051044.s003.tif]

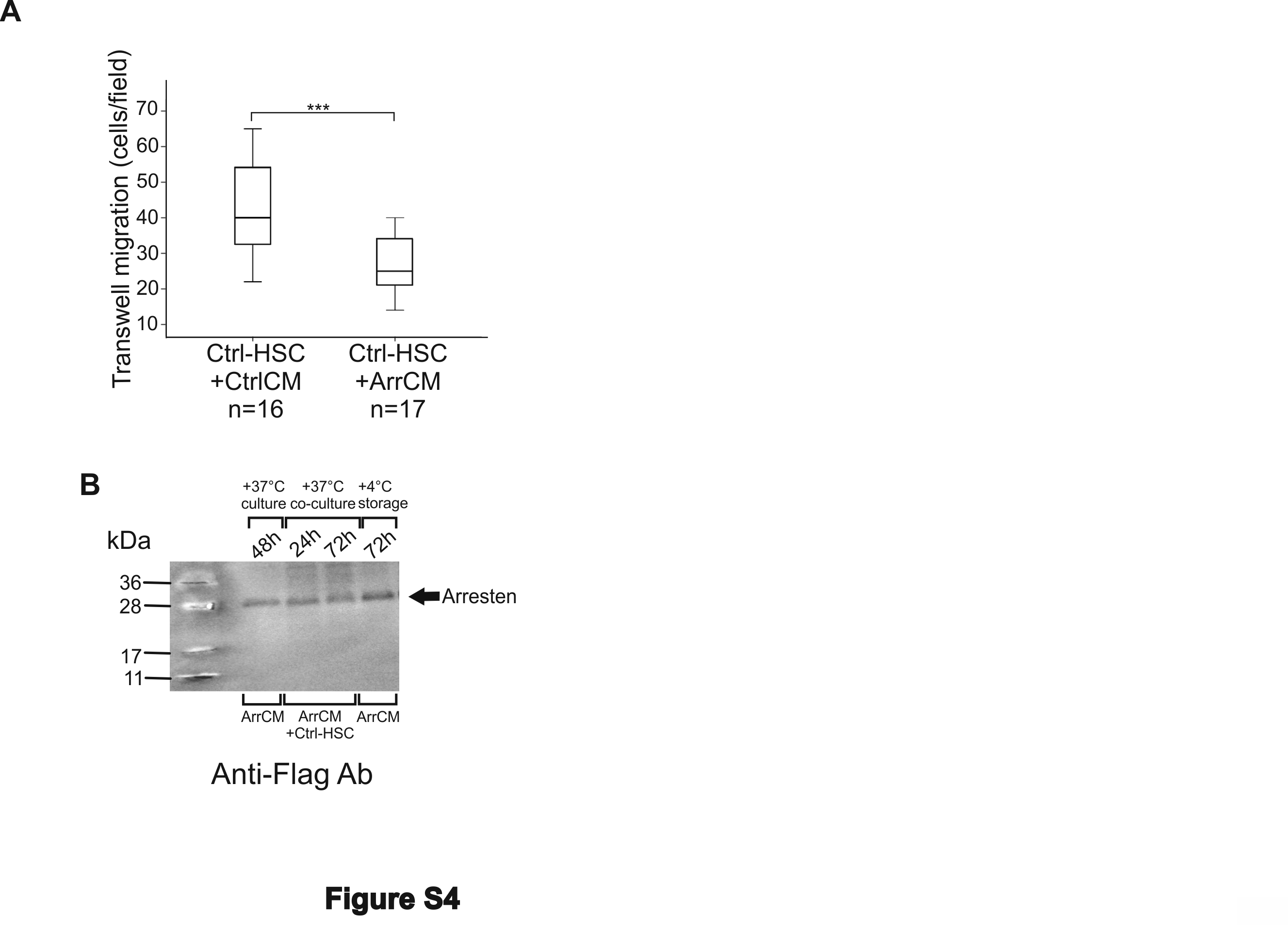

Supplement: Figure S4 — Conditioned Arr-HSC culture medium inhibits HSC-3 cell migration in co-culture experiments. Conditioned media from Ctrl-HSC (CtrlCM) and Arr-HSC (ArrCM) clones were collected at 24 h and administered to Ctrl-HSC cells. A. Cell migration was assayed with a Transwell assay in which 30 000 cells were allowed to migrate through Transwell inserts and the number doing so was counted under a microscope with 50×magnification. Mann-Whitney U-test, ***p<0.001, (n = total number of fields analyzed, 3–5 fields per Transwell insert). B. Recombinant arresten is stable in co-culture at 37°C and in storage at 4°C. The CM was collected from Arr-HSC cells after 48 h culture period. ArrCM was administered to Ctrl-HSC cells and medium samples were collected after 24 h and 72 h incubations at 37°C. A sample of ArrCM stored for72 h at 4°C was also included in the analysis. The CM proteins were concentrated with acetone precipitation and analyzed by Western blotting with an anti-Flag antibody. (TIF) [file pone.0051044.s004.tif]

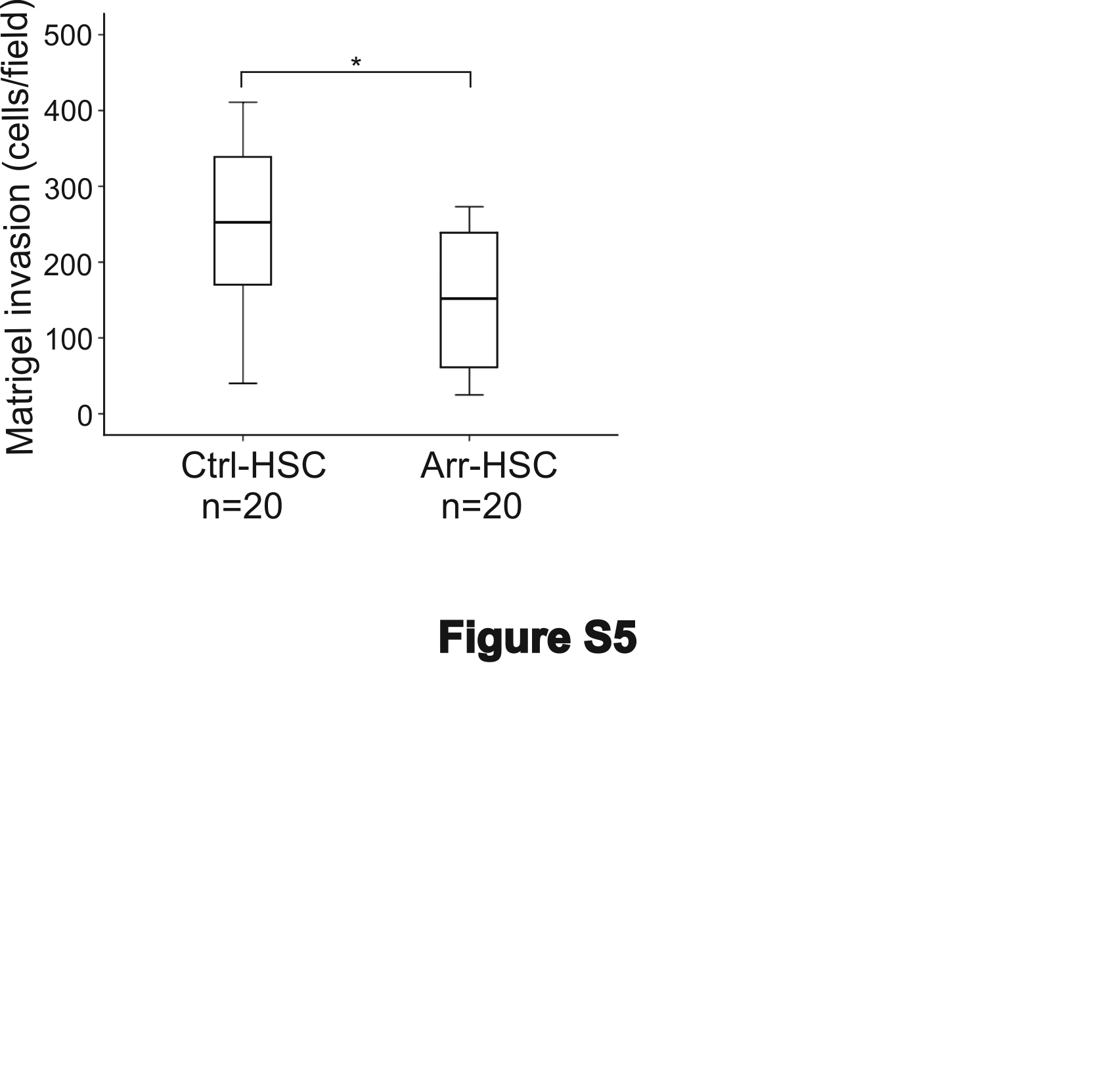

Supplement: Figure S5 — Arresten inhibits cell invasion in vitro . 30 000 Arr-HSC and Ctrl-HSC cells were allowed to invade through the Matrigel-coated Transwell inserts for 22 hours. The invaded cells were stained with hematoxylin and counted under a microscope with 20×magnification. Mann-Whitney U-test, *p<0.05, (n = total number of fields analyzed, 3–5 fields per Transwell insert). (TIF) [file pone.0051044.s005.tif]

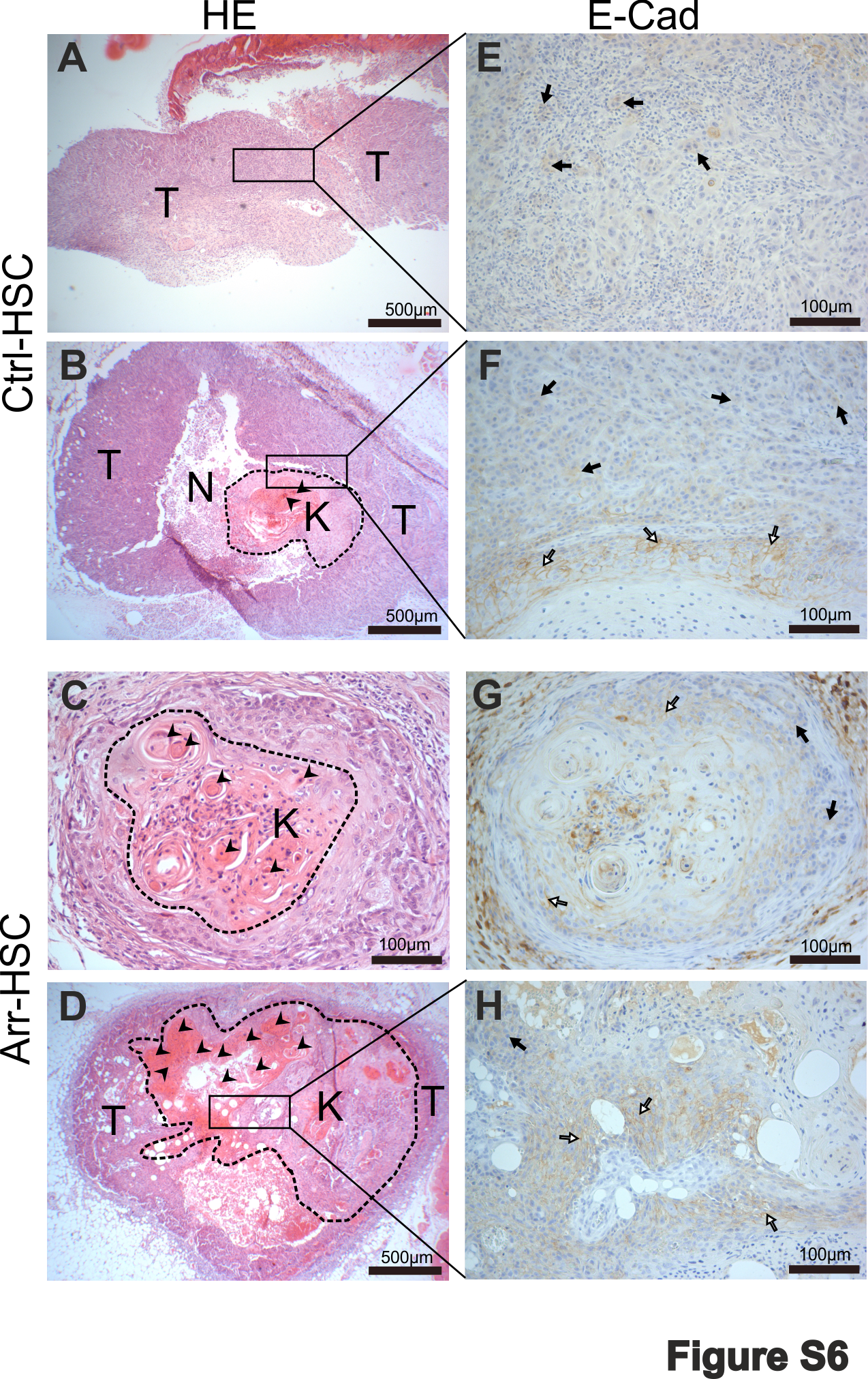

Supplement: Figure S6 — Arresten alters the tissue architecture of HSC-3 xenografts. One million Ctrl-HSC and Arr-HSC cells were injected subcutaneously into the flanks of nude mice (n = 10 per group) and tumor growth was monitored over 16 days. Representative hematoxylin-eosin and E-cadherin stainings of HSC-3 xenografts. A–B. Ctrl-HSC xenografts show an appearance of poorly differentiated squamous cell carcinomas (A). Some tumors contain also keratinized areas (dotted line) in central tumor area (B). C–D. Arr-HSC xenografts resemble moderately or well differentiated squamous cell carcinomas, and relative to the Ctrl-HSC tumors, show more pronounced keratinized areas (dotted line) and keratin pearls (arrowhead), sometimes surrounded by a thin layer of poorly differentiated cells (D). Scale bar 500 µm (A, B, D) and 100 µm (C). E–H. Immonostaining for E-cadherin (brown) showed either diffuse cytoplasmic signals in the poorly differentiated tumor areas (arrow), or membranous staining (open arrow) within the keratinized areas in all xenografts. Scale bar 100 µm. T = tumor; K = keratinized area; N = necrosis. (TIF) [file pone.0051044.s006.tif]

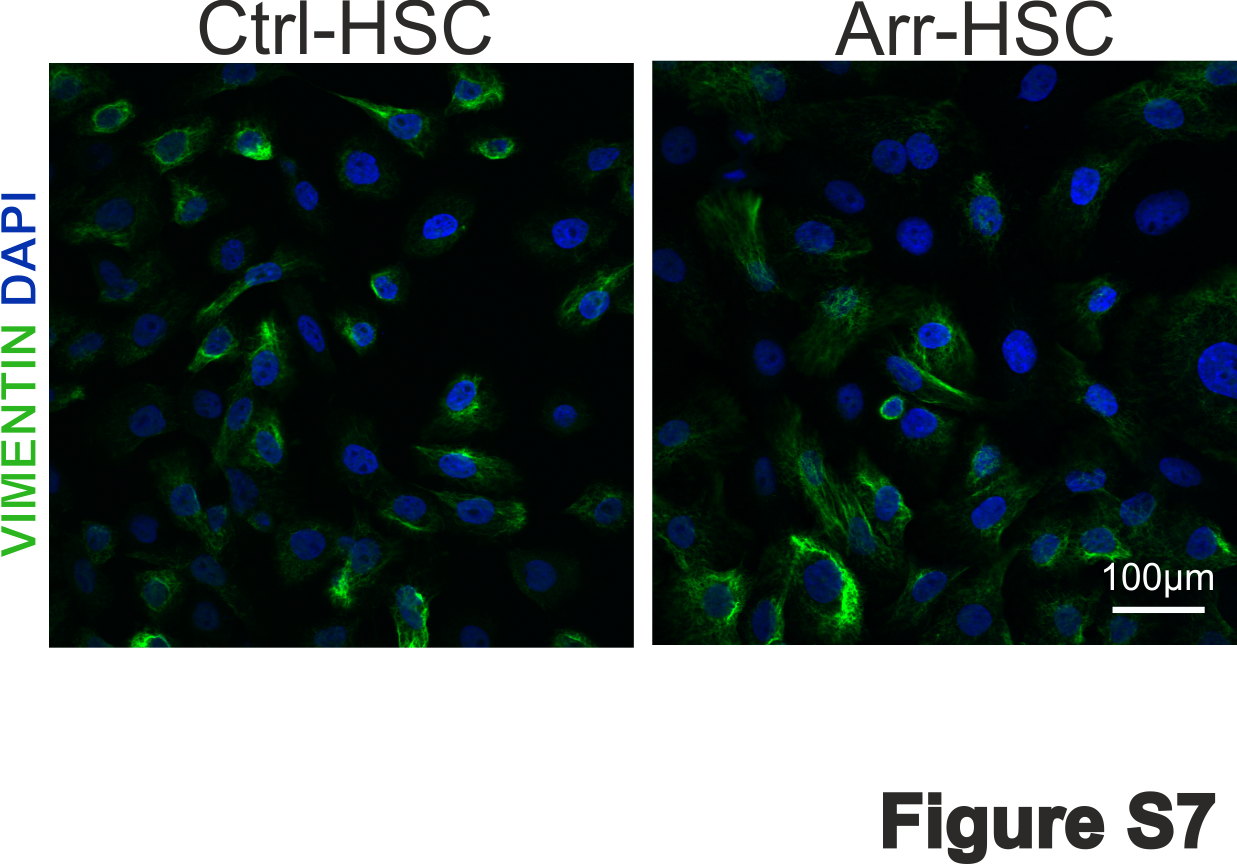

Supplement: Figure S7 — No differences in vimentin staining between Arr-HSC and Ctrl-HSC cells. Immunostaining of vimentin (green) in cultured Ctrl-HSC and Arr-HSC cells (blue, DAPI). Scale bar 100 µm. (TIF) [file pone.0051044.s007.tif]

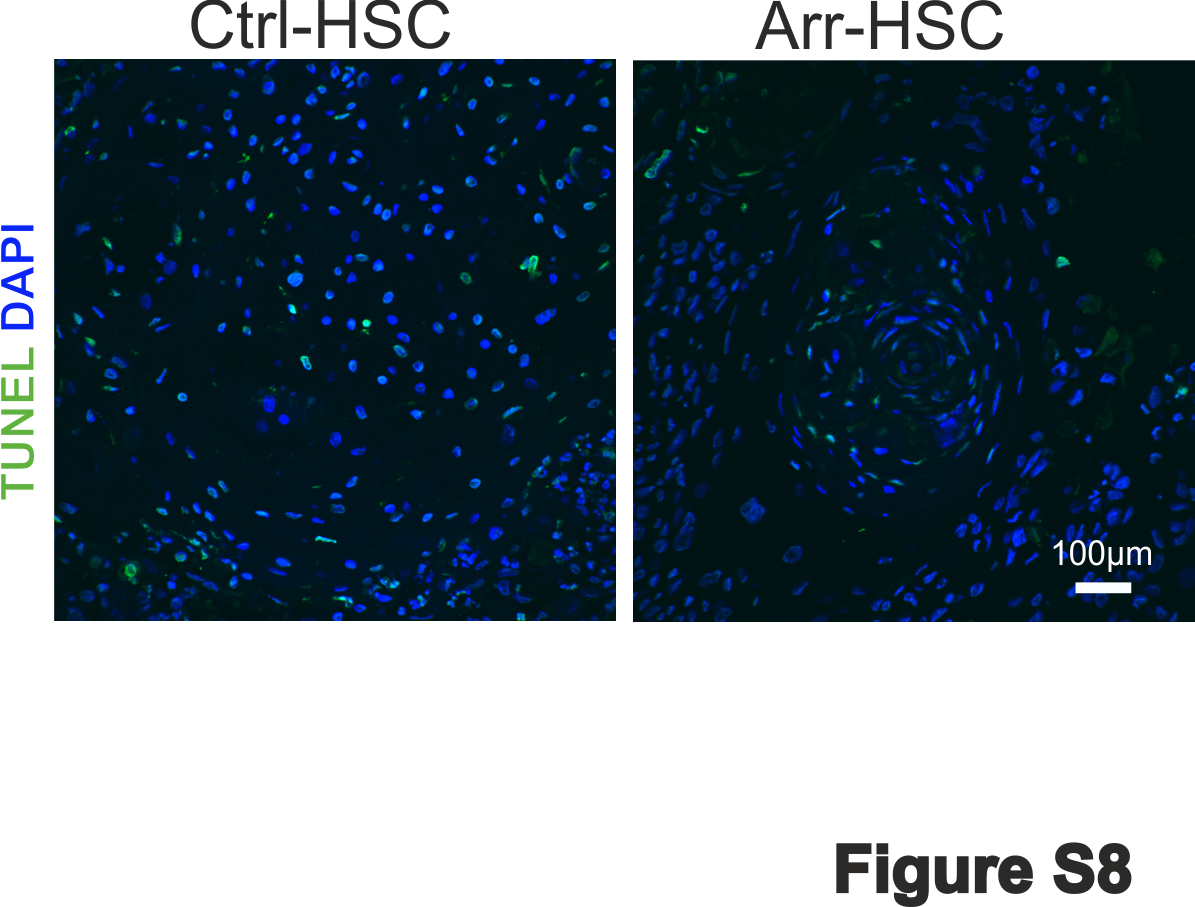

Supplement: Figure S8 — Tunel-positive cells were detected in keratinized or necrotic areas in HSC-3 xenografts. Apoptotic cells were detected by TUNEL assay (green) in HSC-3 xenografts (blue, DAPI). Scale bar 100 µm. (TIF) [file pone.0051044.s008.tif]

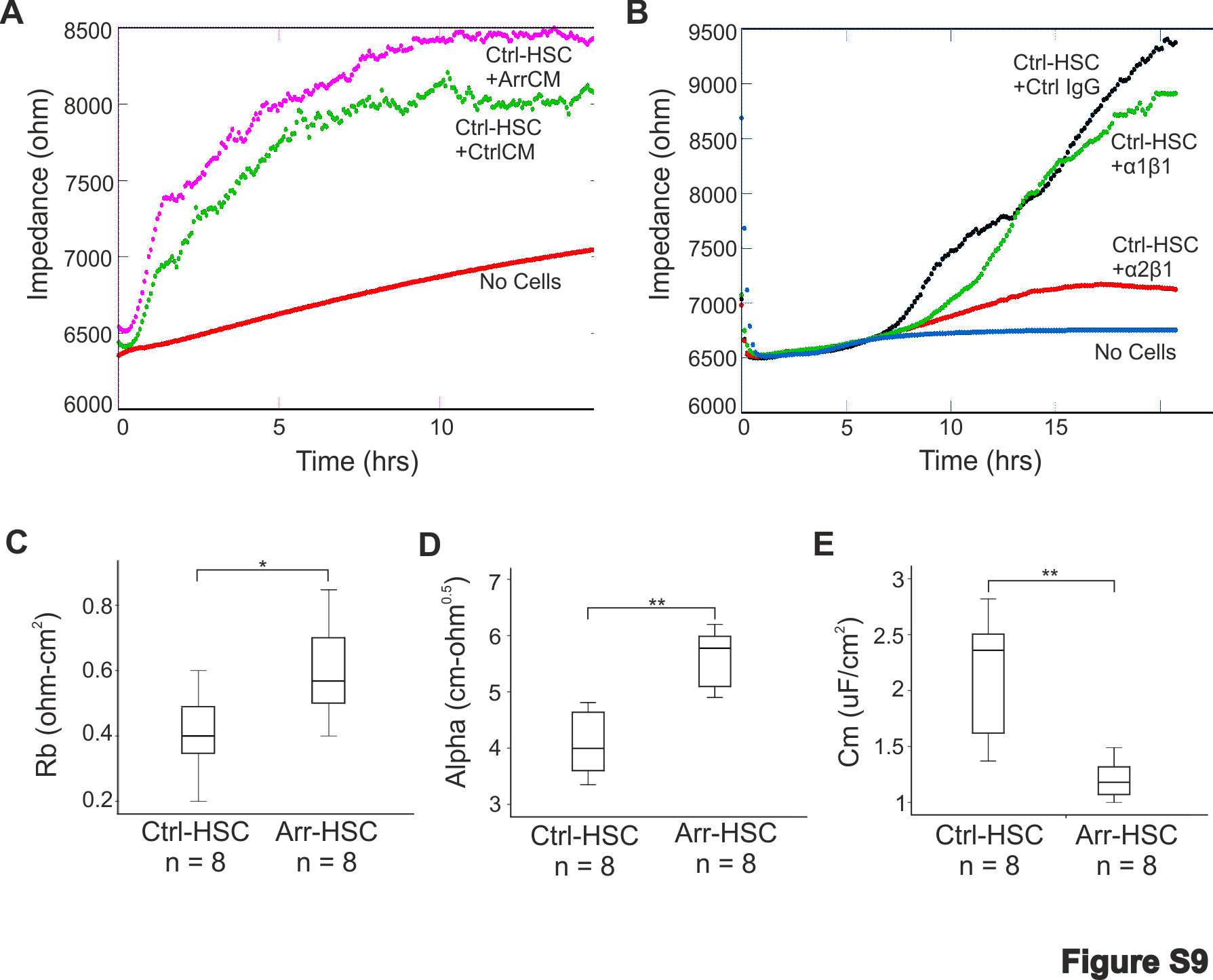

Supplement: Figure S9 — Cell-cell and cell-substrate interactions, and cell membrane capacitance show changes between Arr-HSC and Ctrl-HSC cells. A. The impedance, reflecting cell adhesion and spreading, was measured for Ctrl-HSC cells treated with ArrCM or CtrlCM using electric cell-substrate impedance sensing (ECIS) (mean of duplicate wells of representative ECIS plates). HSC-3 cells treated with ArrCM showed higher impedance than those treated with CtrlCM. B. Ctrl-HSC cells showed reduced spreading in the presence of integrin α2 antibody while control IgG and α1 antibody had no effect on impedance. C–E. A mathematical ECIS™ model of the impedance changes was used to refine the ECIS data and to calculate cell morphological parameters. The barrier function of the cell layer, Rb (C), and the spacing between the cell and the substratum, α (D), were significantly higher in Arr-HSC than in Ctrl-HSC cells. Mann-Whitney U-test, **p<0.01, *p<0.05. E. The cell membrane capacitance, Cm, was significantly decreased in Arr-HSC cells in comparison with Ctrl-HSC cells. Mann-Whitney U-test, **p<0.01. (n = number of ECIS wells). (TIF) [file pone.0051044.s009.tif]
