# Supplementary material for: Arresten, a Collagen-Derived Angiogenesis Inhibitor, Suppresses Invasion of Squamous Cell Carcinoma
Source: PLoS One. 2012 Dec 5;7(12):e51044. doi: 10.1371/journal.pone.0051044 (PMC3515547; doi:10.1371/journal.pone.0051044)
Supplement: Table S1 — Relative mRNA expression of arresten and E-cadherin in the HSC-3 and MDA-MB-435 clones. (DOC) [file pone.0051044.s010.doc]

**Table S1.**

**Relative mRNA expression of arresten and E-cadherin in the HSC-3 and MDA-MB-435 clones*.**

| ***Cell clone*** | ***Arresten*** | ***E-cadherin*** |
| --- | --- | --- |
| **Ctrl-HSC**  N = 6**  n = 12^§^ | 1.3 ± 0.2 | 1.0 ± 0.05 |
| **Arr-HSC**  N = 3  n = 6 | 34.9 ± 1.4*** | 1.9 ± 0.06*** |
| **Ctrl-MDA**  N = 1  n = 3 | 1.1 ± 0.3 | nd^¤^ |
| **Arr-MDA**  N = 2  n = 6 | 1429 ± 66.5*** | nd |

*The expression levels were normalized to that of the GAPDH housekeeping gene and are presented relative to values obtained for Ctrl-cells (means ± SEM);

**N, number of clones analyzed;

^§^n, number of samples analyzed;

***, Students t-test, p< 0.001

^¤^nd, not detected
