# Supplementary material for: Arresten, a Collagen-Derived Angiogenesis Inhibitor, Suppresses Invasion of Squamous Cell Carcinoma
Source: PLoS One. 2012 Dec 5;7(12):e51044. doi: 10.1371/journal.pone.0051044 (PMC3515547; doi:10.1371/journal.pone.0051044)
Supplement: Text S1 — Supplemental methods. Cell culture, cloning of arresten into pcDNA3.1 eukaryotic expression vector, transfection and selection of HSC-3 and MDA-MB-435 cells expressing arresten, purification of recombinant arresten, quantitative real-time RT-PCR (qPCR), cell proliferation assay, MTT viability assay, Transwell invasion assay, E-cadherin staining and apoptosis assay for HSC-3 xenografts and Western blotting. (DOC) [file pone.0051044.s011.doc]

**Text S1.** S**UPPLEMENTAL METHODS**

**ARRESTEN, A COLLAGEN-DERIVED ANGIOGENESIS INHIBITOR, SUPPRESSES INVASION OF SQUAMOUS CELL CARCINOMA**

Mari Aikio, Ilkka Alahuhta, Sini Nurmenniemi, Juho Suojanen, Riitta Palovuori, Susanna Teppo, Timo Sorsa, Carlos López-Otín, Taina Pihlajaniemi, Tuula Salo, Ritva Heljasvaara and Pia Nyberg

*Cell culture*

HSC-3 human tongue squamous carcinoma cells (JCRB, Osaka National Institute of Health Sciences) were cultured in a 1:1 DMEM/F-12 medium (Invitrogen) supplemented with 100 U/ml penicillin, 100 µg/ml streptomycin, 250 ng/ml fungizone, 50 µg/ml ascorbic acid and 0.4 µg/ml hydrocortisone (all from Sigma-Aldrich) and 10% heat inactivated fetal bovine serum (FBS; Perbio Science). MDA-MB-435 human breast carcinoma cells (ATCC) [1] were cultured in DMEM high-glucose supplemented with 10% FBS, 100 U/ml penicillin and 100 µg/ml streptomycin. The transfected cell lines were cultured under selective pressure in the presence of 300 or 1000 µg/ml of Geneticin G418 antibiotic (Invitrogen). Primary human gingival fibroblasts were obtained from biopsies of healthy gingiva [2], and cultured in DMEM supplemented with 100 U/ml penicillin, 100 µg/ml streptomycin, 50 µg/ml ascorbic acid, 250 ng/ml fungizone, 1 mM sodium pyruvate (Sigma-Aldrich) and 10% FBS (Perbio Science). All the cell lines were used at low passages.

*Cloning of arresten into pcDNA3.1 eukaryotic expression vector*

The human arresten construct (a kind gift from Raghu Kalluri, Beth Israel Deaconess Medical Center, Harvard Medical School, Boston, MA) was used as a template to clone arresten into the pcDNA3.1 eukaryotic expression vector (Invitrogen), thus enabling protein production in mammalian cells and providing resistance to the selection antibiotic. The full-length arresten was amplified by PCR from the original arresten construct [3, 4] using the following primers: forward primer containing the *EcoR I* site (underlined) and BM40 signal sequence (italics) AATGCCGAATTC*ATGAGGGCCTGGATCTTCTTTCTCCTTTGCCTGGCCGGGAGGGCTCTGGCAGCCCCACTT*TCTGTTGATCACGGCTTCCTTG and reverse primer containing Flag tag (italics) and the *Xho I* restriction site (underlined) AGCGCCCTCGAGTTA*CTTATCGTCGTCATCCTTGTAATC*TGTTCTTCTCATACAGACTTGGCAGCGGCTGACGTGCGTGCGCA. PCR was performed with 0.4 U Phusion Hot Start polymerase (Finnzymes), 200 µM dNTP (Promega), 1 µM of each primer, 3% DMSO and 200 ng plasmid template in a total volume of 20 µl of 1× Phusion HF buffer. After initial denaturation (2 min at 98ºC), 35 cycles were performed (30 sec at 98ºC, 30 sec at 60ºC and 30 sec at 72ºC) followed by a final extension step. The resulting PCR fragment and the pcDNA3.1 vector were digested with *EcoR I* (New England Biolabs) and *Xho I* (New England Biolabs) and ligated together using 1 U of T4 DNA ligase (Promega). The resulting ligation reactions were transformed into OneShot competent *E.coli* cells (Invitrogen) and the plasmids were purified with the PureYield Plasmid Midiprep System (Promega). The presence of the insert and the correct sequence was verified by restriction analysis and sequencing (ABI PRISM-3100 sequencer).

*Transfection and selection of HSC-3 and MDA-MB-435 cells expressing arresten*

HSC-3 and MDA-MB-435 cells were transfected with human arresten cDNA, or with an empty pcDNA3.1 vector using either the Lipofectamine plus (Invitrogen) or Fugene HD (Roche Molecular Biochemicals) transfection reagents as recommended by the manufacturers. The transfected cells were selected with 300 µg/ml (HSC-3) or 1000 µg/ml (MDA-MB-435) of Geneticin G418 antibiotic (Invitrogen) to obtain stable populations of cells expressing human arresten. The clones used in the experiments were named Ctrl-HSC, Arr-HSC(1), Arr-HSC(2), Ctrl-MDA, Arr-MDA(1) and Arr-MDA(2).

*Purification of recombinant arresten*

For purification of recombinant arresten we used HEK 293 cell line that had been stably transfected with the arresten plasmid by Fugene reagent (Roche). Transfected HEK-cells produced and secreted arresten into the culture media the presence of Geneticin selection antibiotic. The media were collected twice a week and a protease inhibitor (Roche) was added to prevent proteases to cleave arresten molecules. The arresten containing media was run through a gel affinity chromatography column (ANTI-FLAG^R^ M2 Affinity Gel, Sigma-Aldrich). Glycine was used to elute arresten from the column and 2 ml fractions were collected. The pH of the fractions was adjusted by 50 µl 1 M Tris-HCl (pH 8). The fractions were dialyzed and concentrated with Ultra tubes (Millipore) at 3900 rpm at 4^o^C for 10 minutes. Protein concentration was measured with BCA^TM^ Protein Assay Kit (Pierce).

*Quantitative real-time RT-PCR (qPCR)*

Total RNA was isolated from cells using the E.Z.N.A.™ total RNA kit (Omega). 1 µg of RNA was used for cDNA synthesis with the iScript™ cDNA synthesis kit (BioRad). mRNA expression levels were analysed by qPCR using gene-specific primers [human glyceraldehyde-3-phosphate dehydrogenase (GAPDH) 5′TGCACCACCAACTGCTTAGC-3′ and 5′GGCATGGACTGTGGTCATGAG-3′; human E-cadherin 5′GAACGCATTGCCACATACAC -3′ and 5′ATTCGGGCTTGTTGTCATTC -3′; human arresten 5′CTCTACGTGCAAGGCAATGA-3′ and 5′-GAAGGGCATTGTGCTGAACT-3′], iTaq™ SYBR® Green Supermix with ROX (Bio-Rad) and the MX3005 (Stratagene) equipment. Relative gene expression levels were calculated by the 2^ΔΔCt^ method [5] and normalized to RNA of the GAPDH housekeeping gene. All data were expressed relative to values obtained for mock-transfected cells.

*Cell proliferation assay*

Cell proliferation was measured using the colorimetric Cell Proliferation ELISA BrdU kit (Roche) according to the manufacturer’s instructions. Briefly, 5000 Ctrl-HSC and Arr-HSC cells were allowed to attach to 96-well plates. After 2 hours the medium was replaced with a serum-free medium and the cells were left to grow overnight. Next day the cells were allowed to grow in either serum-free or serum-containing medium for 24 hours. They were labeled with 1:100 BrdU labeling reagent. After 3-h incubation they were fixed with 200 µl FixDenat reagent for 30 min at room temperature. The FixDenat solution was then removed and 100 µl of 1:100 BrdU antibody dilution was added and the plates were incubated for 1.5 hours at room temperature and washed three times with 200 µl washing solution before 100 µl substrate regent was added. After 30 min of incubation 25 µl of HS_2_SO_4_ was used to stop the reaction. Absorbance was measured with an ELISA reader (BioRad) at 450 nm.

*MTT viability assay*

Cell viability was measured by means of an MTT [3-(4,5-dimethylthiazol-2-yl)-2,5-diphenyl tetrasodium bromide] assay, which is based on the formation of black crystals by the mitochondria of viable cells in the presence of MTT. 5000 Ctrl-HSC and Arr-HSC cells were grown on 96-well plates with 100 µl HSC-3 medium and their viability was measured both in serum-free and serum-containing media. The cells were left to grow for 20, 44 or 68 hours. 10 µl of MTT reagent was incubated with the cells for 4 hours and the crystals were dissolved in 100 µl DMSO and their absorbance measured with an ELISA reader (BioRad) at 540nm.

*Transwell invasion assay*

The invasion assays were performed on 24-well plates using ThinCerts of pore size 8 μm (Greiner Bio-One) coated with 20 µg of ECL cell attachment matrix (Millipore). Briefly, the coated inserts were hydrated with warm DMEM without serum in 5% CO_2_ at 37°C for two hours. A total of 5 × 10^4^ Ctrl-HSC and Arr-HSC cells were seeded into the upper chamber containing DMEM without serum, and the lower chamber contained DMEM with 10% FBS. The cells were incubated at 37°C in 5% CO_2_ for 22 h. Non-invading cells were removed from the upper membrane by scrubbing with a cotton swab, and the invading cells were fixed with 100% methanol at -20°C for five minutes, stained with hematoxylin solution (Sigma Aldrich), and visualized under a light microscope (Leica). The number of stained cells was counted in five microscope fields (20× magnification).

*E-cadherin staining and apoptosis assay for HSC-3 xenografts*

For immunohistochemistry of E-cadherin (Cell Signaling Technology), sections were stained using the REAL EnVision Detection System (Dako) according to the manufacturer´s instructions; the primary antibody (Cell Signalling Technology) was incubated at 37°C for 1 h , after which the secondary antibody was applied for 30 min. The presence of the antigen was visualized using DAB Peroxidase Substrate (Vector) for 3 min and counterstained. In the negative controls normal serum or IgG of the appropriate species (Dako) was used instead of the primary antibody. TUNEL assay was used to quantify the amount of apoptotic cells in HSC-3 xenografts. The apoptotic cells were labeled according to the instructions of the *In Situ* Cell Death Detection Kit (Roche). Confocal images were captured using a laser confocal microscope (Olympus IX81).

*Western blotting*

Serum-free conditioned media were concentrated 1:4 with Amicon Ultra concentration tubes (Millipore) and analyzed by Western blotting. Alternatively, CM were concentrated with acetone precipitation. Cell extracts were prepared for Western blotting by scraping the cells into lysis buffer (50 mM Tris, 10 mM CaCl_2_, 150 mM NaCl, 0.05% Brij, pH 7.5) on ice, rotating the mixture at 4ºC for 4 h and collecting the supernatant by centrifugation at 12000×g for 10 min. For Western blotting of the signaling molecules Bax and Bcl-xL, cell extracts were prepared in 1× lysis buffer (Cell Signaling Technology) according to [4]. Briefly, the Western blot samples were separated by 12% SDS-PAGE under reducing conditions and transferred to Immobilon-P PVDF membranes (Millipore). Non-specific binding was blocked with 5% non-fat dry milk for 1 h. The membranes were probed with polyclonal rabbit anti-E-cadherin (1:50; Cell Signaling Technology), polyclonal rabbit anti-Bax (1:1000; Cell Signaling Technology), polyclonal rabbit anti-Bcl-xL (1:1000; Cell Signaling Technology), monoclonal mouse anti-Bcl-2 (1:1000; clone 124; Dako), and monoclonal mouse anti-Flag-M2-tag (1:1000; Sigma Aldrich) overnight followed by a biotinylated secondary antibody (Dako). The immunoreactive proteins were visualized with Vectastain Elite ABC Kit detection reagents (Vector) according to the manufacturer’s instructions. β-actin (Abcam) was used as a loading control.

**SUPPLEMENTAL REFERENCES**

[1] Chambers AF**.** MDA-MB-435 and M14 cell lines: identical but not M14 melanoma? (2009) Cancer Res 69: 5292-5293.

[2] Kylmäniemi M, Oikarinen A, Oikarinen K, Salo T. (1996) Effects of dexamethasone and cell proliferation on the expression of matrix metalloproteinases in human mucosal normal and malignant cells. J Dent Res 75: 919-926.

[3] Colorado PC, Torre A, Kamphaus G, Maeshima Y, Hopfer H, et al. (2000) Anti-angiogenic cues from vascular basement membrane collagen. Cancer Res 60: 2520-2526.

[4] Nyberg P, Xie L, Sugimoto H, Colorado P, Sund M, et al*.* (2008) Characterization of the anti-angiogenic properties of arresten, an alpha1beta1 integrin-dependent collagen-derived tumor suppressor. Exp Cell Res 314: 3292-3305.

[5] Livak KJ, Schmittgen TD. (2001) Analysis of relative gene expression data using real-time quantitative PCR and the 2(-Delta Delta C(T)) Method 25: 402-408.
